# Effects of Cultured Root and Soil Microbial Communities on the Disease of *Nicotiana tabacum* Caused by *Phytophthora nicotianae*

**DOI:** 10.3389/fmicb.2020.00929

**Published:** 2020-05-15

**Authors:** Tianbo Liu, Yunhua Xiao, Jian Yin, Tuyong Yi, Zhicheng Zhou, Tom Hsiang, Qianjun Tang, Wu Chen

**Affiliations:** ^1^College of Plant Protection, Hunan Agricultural University, Changsha, China; ^2^Central South Agricultural Test Station of China Tobacco, Changsha, China; ^3^College of Bioscience and Biotechnology, Hunan Agricultural University, Changsha, China; ^4^School of Environmental Sciences, University of Guelph, Guelph, ON, Canada

**Keywords:** black shank, root functional microflora, soil functional microflora, diversity indices, functional strains

## Abstract

**Background:**

Black shank, caused by the oomycete pathogen *Phytophthora nicotianae*, is responsible for huge economic losses worldwide. Research has focused on biocontrol to prevent disease and to minimize the use of synthetic fungicides.

**Methods:**

We explored and compared the efficacy of suppressive microflora cultured from soil and roots on the growth of *P. nicotianae* for controlling the incidence of black shank.

**Results:**

We found that 31 microfloral communities, enriched from 40 root samples but only 18 microfloral communities from soil samples, were antagonistic to *P. nicotianae.* In the field experiment, the root functional microflora (RFM) showed a greater suppressiveness of black shank than the soil functional microflora (SFM), while both RFM and SFM altered diversity, composition, structure, and interaction of soil bacterial communities during plant growth. Although the inoculation of RFM onto roots significantly (*p* < 0.05) decreased microbial diversity, molecular ecological network analysis indicated more possible interactions among soil microbes, while an opposite trend was observed with SFM inoculation. Linear regression analysis revealed that diversity indices were negatively correlated with suppression on the black shank, suggesting that specific taxa (e.g., OTU_322 and OTU_6478) could colonize and be active during plant growth at the expense of microbial diversity. In addition, 18 functional strains, isolated and screened from 3 RMF (12 strains) and 3 SMF (6 strains), were identified as bacterial genera *Acinetobacter* (12), *Enterobacter* (1), *Bacillus* (1), *Stenotrophomonas* (2), and *Citrobacter* (2). Spearman’s ranked correlation tests revealed that relative abundances of some OTUs affiliated with genera *Acinetobacter*, *Enterobacter*, and *Bacillus* were significantly (*p* < 0.05) and positively correlated with the level of disease suppression.

**Conclusion:**

Microfloral communities or key functional species isolated from plant roots might be more effective in controlling black shank than those from soil, and they may be developed for disease control.

## Introduction

Black shank, caused by the oomycete pathogen *Phytophthora nicotianae*, is one of the primary diseases of *Nicotiana tabacum*, and a global concern resulting in huge economic losses (Lucas, 1975). Because the pathogen can survive in the soil and plant tissues at all stages during growth, black shank is one of the most difficult diseases to control. Soil environments, including soil type, nutrients, and populations of bacteria and fungi, are dominant factors for black shank occurrence and development (Kyselková et al., 2009). The traditional approaches to control *N. tabacum* diseases generally include synthetic fungicides (You et al., 2016), soil removal and replacement, crop rotation (Niu et al., 2017), and/or disease-resistant varieties (Deb et al., 2018). However, many disadvantages have been found during the use of those traditional approaches, such as the emergence of fungicide-resistant populations, disruption of local ecosystems, large labor requirements, and threats to human health. As such, it may be better to use several control methods simultaneously (Stumbriene et al., 2018). Therefore, it is urgent to develop effective and environmentally friendly methods to control *N. tabacum* diseases.

The mechanisms of resistance to plant diseases are complex (Deverall, 1987). Secretions of plants, e.g., cutin (Maurel et al., 1994), and microbial communities in the rhizosphere soil and on plant surfaces (Kwak et al., 2018) are the first protective screen against plant pathogens (Weyens et al., 2009). The plant immune systems (Jones and Dangl, 2006) can further limit microbial growth. For plant autoimmune mechanisms, plants can recognize pathogen-associated molecular patterns and pathogen-delivered effectors, and stimulate responses. Plants can strengthen their existing defenses, and also can elicit other defense mechanisms (Gómez-Gómez and Boller, 2002) mediated by signaling mechanisms involving several secondary signal molecules, such as salicylic acid, ethylene, and jasmonic acid to fine-tune the defense response (Wang et al., 2002). However, the plant immune systems are determined by plant genes (Deverall, 1987), which is difficult to manipulate directly. Therefore, attention has been paid toward endophytes and other associated microorganisms of plants, to develop biological control methods using beneficial antagonists as promising choices.

In recent years, many microbial ecological agents have been isolated from plant tissues and soil samples to control plant diseases. *Bacillus* spp., isolated from wheat roots and soil samples, could effectively reduce the growth of *Fusarium graminearum* strains causing Fusarium head blight (Stumbriene et al., 2018); *Bacillus subtilis* Tpb55, isolated from *N. tabacum* leaves, could effectively control the hyphal growth of *P. nicotianae* and had an obvious suppressive effect on black shank (Han et al., 2016); *Flavobacterium* sp. TRM1, isolated from rhizosphere soil, could suppress *Ralstonia solanacearum* disease development (Kwak et al., 2018); *Pseudomonas aeruginaosa* NXHG29, isolated from soil (Li et al., 2012), could decrease the incidence of *N. tabacum* bacterial wilt and black shank (Ma et al., 2018). Kloeppe et al. (1999) suggested that plant growth-promoting rhizobacteria, endophytic bacteria, and indigenous soil bacterial communities could be used to effectively decrease crop mortality.

Besides, the interaction of many microorganisms may be essential for plants to suppress disease incidence (Miliute et al., 2015). Compared with individual organisms as biocontrol targets, microflora may be better as they can more easily colonize a new environment (Delgado-Baquerizo et al., 2016; Smith et al., 2018). We aimed to understand:(i) the effect of functional root and soil microflora on suppressing black shank incidence; (ii) the effect of inoculations of microflora on general soil bacterial communities; and (iii) isolation and characterization of individual strains and their effects on black shank incidence. To achieve those goals, we inoculated functional microbial communities enriched from tobacco roots and rhizosphere soils onto roots during the stage of tobacco seedlings, isolated several functional strains with antifungal activity, and monitored microbial community succession by metagenomic sequencing of 16S rRNA gene amplicons and other techniques.

## Materials and Methods

### Culture and Screening of Functional Microflora

Microflora were enriched from tobacco (*Nicotiana tabacum* plants cultivar K326) rhizosphere soil and root samples obtained from a long-term ecological research site located in Xiangxi, Hunan, China. *N. tabacum* cultivar K326 was introduced from Northup King Seed Company (America) to Yunnan province (China) in 1985, and is now preserved in Tobacco Research Institute, Chinese Academy of Agricultural Sciences, where we obtained the seeds and used it with permission. Forty rhizosphere soil samples (DP_S1-DP_S20; HP_S1-HP_S20) and forty root samples (DP_R1-DP_R20; HP_R1-HP_R20) from 20 diseased plants (DP) and 20 healthy plants (HP) were collected using a clean spade at the *N. tabacum* harvesting stage in July 2017. Rhizosphere soils were collected following Kwak et al. (2018). All samples were refrigerated until use within 3 days. Because of tight binding to roots, rhizosphere soils were separated by vigorous vortexing in sterile ddH_2_O. To culture the rhizosphere soil microflora, these soil solutions were incubated in a half-strength LB Broth Medium (pH 7.2) for 24 h at 25°C and 150 rpm. These seed cultures were then stored at -20°C until use.

The endophytic root microflora were cultured following Geisen et al. (2017). The collected roots were thoroughly and successively washed with sterile ddH_2_O, 3 s in 75% ethanol, and washed again with sterile ddH_2_O. Then, the roots were cut to approximately 0.5 cm using sterilized scissors and were incubated in a half-strength LB Broth Medium for 24 h as above. These seed cultures were then stored at -20°C until use.

In order to enrich the functional microflora for black shank control, the antagonism experiments were conducted using the pathogen *Phytophthora nicotianae* HD1 obtained originally from diseased *N. tabacum* roots. The pathogen inoculum (5 mm diameter PDA plugs) was placed in the center of each 9 cm diameter PDA plate. Two microliters were taken from each frozen stock, and grown in 100 mL of half-strength LB broth with shaking at 100 rpm at 25°C for 3 days. An aliquot of 1 mL was taken from each of the 40 cultures and filtered through 0.22 μm filters (Merck Millipore, United States). Two 200 μL droplets of each filtrate were placed on a plate 2 cm away from a pathogen plug following Stumbriene et al. (2018). Antagonism was assessed by examining the inhibition of pathogen growth as it approached the microfloral culture filtrate and the presence of inhibition zones.

### Field Design

To explore and compare the effects of root functional microflora (RFM) and soil functional microflora (SMF) on suppression of black shank, three RFM (R1, R2, and R3) and three SFM (S1, S2, and S3), with the most effective in the above antagonistic test, were selected for the field experiments. Before application, 0.1 mL was taken from each of six microflora frozen seed cultures and fermented at 25°C in 10 L stirred bioreactors (Shanghai NOVA Engineering & Technology Development Co., Ltd, Shanghai, China) containing 7.0 L half-strength LB Broth Medium. The dissolved oxygen concentration was monitored with an on-line probe (Mettler-Toledo Process Analytical Instruments, Zurich, Switzerland). Media were sparged as necessary with filtered air at four air volumes per culture volume per minute. The agitation was controlled between 500 and 1000 rpm to maintain the dissolved oxygen levels at > 20%. After fermentation for 36 h, the final concentration of microbial cultures was about 2 × 10^10^ cells/mL. One milliliter fermentation liquor of each microflora was centrifuged at 10,000 rpm for 2 min, the supernatant was removed, and then the collected microflora was stored at -20°C for DNA extraction, amplification, and 16S rRNA gene amplicon sequencing.

Seven areas (2 m × 10 m) were designed and randomly arranged in a complete block design in Huayuan Planting Base, which is located in Hunan, China. *N. tabacum* cultivar K326 was used as experimental plants in filed experiments. They were transplanted to these seven areas (45 plants in each area) in April, 2018. At the transplanting stage (day 0) and at the resettling stage (day 30), 300 mL fermentative microbial culture (R1, R2, R3, S1, S2, and S3 microflora each at 2 × 10^10^ cells/mL) and 300 mL ddH_2_O (the control group, CK) were irrigated onto the roots of each plant. The agricultural management practices and fertilization regimes were similar in all seven areas described in detail in our previous study (Xiao et al., 2018). No pest or disease controls were applied during the experiment.

### Sampling

Eight samples were collected from each area using the checkerboard sampling method on days 30, 60, and 90. In the checkerboard sampling method, each field was divided into eight areas (each 1 m × 2.5 m), and the central point of each area was the sampling site. Soil samples (10.0 g) beside the roots (5–20 cm depths, 3 cm diameter widths) were obtained and were stored at -80°C before DNA extraction. The blank shank disease index of each area on day 90 was determined based on the Chinese national standard GB/T 23222-2008, as described by Gao et al. (2015), ranging from a low of 0 to a high of 9. Disease suppression was calculated as follows: Suppression (%) = (DI_CK_-DI_X_)/ DI_CK_ × 100, where DI_CK_ represents the disease index of the control group, and DI_X_ represents the disease index of each treatment group.

### DNA Extraction, Amplification, 16S rRNA Gene Sequencing and Data Processing

Microbial community DNA extraction, 16S rRNA gene amplification and sequencing, as well as data processing followed Xiao et al. (2018). The soil from each root collection was homogenized, and 1.0 g from each sample was used for DNA extraction using a PowerSoil DNA Isolation Kit (MO BIO, San Diego, CA, United States). DNA extracts were purified by electrophoresis on a 0.7% agarose gel and extracted using a DNA gel extraction kit (OMEGA, United States). 16S rRNA was amplified with primer pair 515F (5′-GTGCCAGCMGCCGCGGTAA-3′) and 806R (5′-GGACTACHVGGGTWTCTAAT-3′), combined with Illumina adapter sequences, a pad and a linker of two bases, and barcodes on the reverse primer. PCR products were purified by kit as above, and the concentration was quantified with a NanoDrop ND-1000 spectrophotometer (NanoDrop Technologies, Wilmington, NC, United States). The products were sequenced on a MiSeq platform (Illumina, San Diego, CA, United States) using a 500 cycle kit.

### Network Construction and Characterization

Random matrix theory (RMT)-based approaches were used for network construction (Yin et al., 2015), hub and connector OTU identification, and the topological property was determined with a similar threshold (0.960). OTUs, which presented in 8 out of 8 replicates, were used for network analysis to ensure correlation reliability. Various network properties were characterized, such as average degree, average path distance, average clustering coefficient, and modularity index. The network modules were generated using rapid greedy modularity optimization. The experimental data used for constructing phylogenetic molecular ecological networks (pMENs) based on 16S rRNA gene sequencing data, and Cytoscape 3.6.1 software was used to visualize the network graphs. The pMENs were constructed separately based on sequencing data of the nine treatments to reveal the effects of the representative root and soil microflora on the microbial network interactions.

### Statistical Analysis

The community diversity was assessed using the Shannon diversity index (*H*’) and species diversity index. Differences in diversity and relative abundances of bacterial composition based on Tukey’s test were conducted by a one-way analysis of variance (ANOVA) and response ratio following (Deng et al., 2012). Detrended correspondence analysis (DCA) and dissimilarity tests were conducted to compare different bacterial community structures. Linear regression analysis was carried out to explore relationships between the disease suppression and microbial diversity (Barberán et al., 2014; Wagg et al., 2014; Cui et al., 2016). All analyses were performed using R v.3.6.3 and STAMP v 2.1.3.

### Functional Species Isolation and Identification

To obtained functional strains to antagonize *P. nicotianae*, the selected 3 RFM (R1, R2, and R3) and 3 SFM (S1, S2, and S3) cultures were serially diluted and cultivated on a half-strength LB agar. The single species antagonism experiments were also conducted as above. The antagonistic isolates were identified following Sheng et al. (2008) by comparing their 16S rRNA gene sequences to the NCBI database. To investigate whether the antagonistic isolates were colonized in the field experiments, their 16S rRNA gene sequences were also mapped onto the representative sequences of the functional OTUs (affiliated to the same genera based on the phylogeny) among the microbial community data locally. Additionally, Spearman’s ranked correlation tests were carried out to explore relationships between the disease suppression and functional genera/OTUs abundances. Random forest analysis was conducted to evaluate the role and the relative importance of the functional OTUs in the field experiment.

## Results

### Antagonistic Effects on *P. nicotianae* HD1 of Cultured Soil and Root Microflora

To enrich functional microflora, rhizosphere soil and root samples were cultured, and antagonism experiments were done in the lab and the field. The antagonism results showed that some microfloral community enrichments could inhibit the growth of *P. nicotianae* HD1 (Supplementary Table S1), including 10 DP_S, 8 HP_S, 15 DP_R, and 16 HP_R. It indicated that the number of antagonistic microfloral cultures from soil was significantly (*p* < 0.05) lower than that from roots. Among these 18 SFM and 31 RFM community cultures, the ones showing the highest antagonism in the root or soil groups in dual plate cultures were selected for field experiments. These included three SFM (HP_S10, DP_S14, and HP_S12) and three RFM (DP_R12, DP_R5, and HP_R2) to examine their effects on inhibiting black shank incidence. In order to make it clearer, HP-S10, DP_S14, HP_S12, DP_R12, DP_R5, and HP_R2 were renamed as S1, S2, S3, R1, R2, and R3, respectively.

### Disease Indices and Suppression of Black Shank With Soil and Root Microflora

**FIGURE 1:**
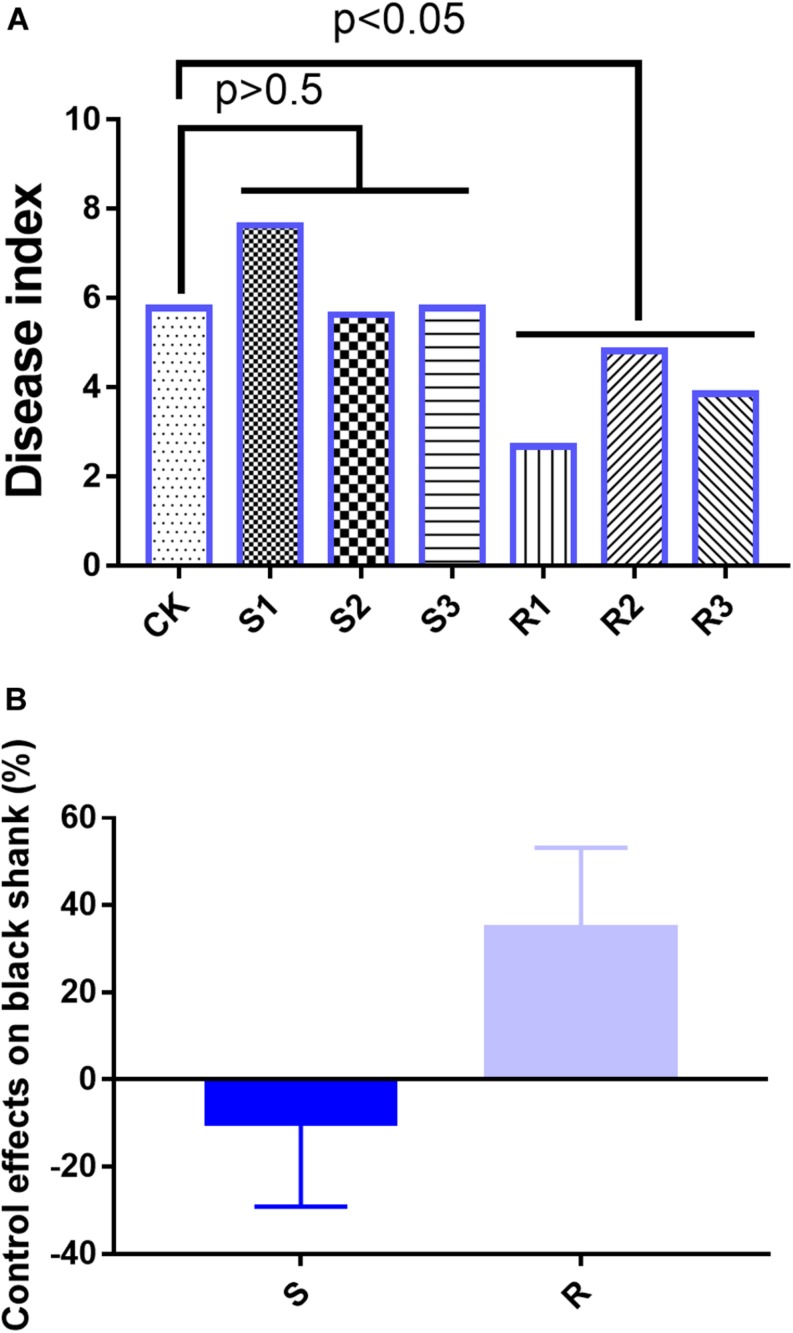
**(A)** Disease index and **(B)** suppression of black shank. CK, control group; R, roots with three replicate samples; S, soil with three replicate samples. The soil samples taken together were not significantly different from the control, where the root samples were different at *p* = 0.05.

In the field experiments, on day 90, the disease indices were 5.78 (CK), 7.63 (S1), 5.63 (S2), 5.77 (S3), 2.67 (R1), 4.81 (R2), and 3.85 (R3) (Figure 1A). Compared with the CK group, the average disease index in the group inoculated with SFM showed no significant differences, but the average disease index in the group inoculated with RFM showed significant (*p* < 0.05) differences (Figure 1A). Disease suppression levels were -32.1% (S1), 2.6% (S2), 0.1% (S3), 53.85% (R1), 16.67% (R2), and 33.33% (R3), which indicated that RFM were more effective than SFM in reducing the incidence of black shank (Figure 1B).

### Overview and Comparison of the Cultured Functional Soil and Root Microfloral Composition

All the 16S rRNA gene sequences have been submitted to the Sequence Read Archive (SRA) of NCBI, and the accession numbers are SUB5647083 and SUB5639076. After clustering at 97% sequence identity, 772 OTUs were identified to comprise the cultured microflora. DCA results showed that the samples in the R group segregated from the samples in the S group (Supplementary Figure S1). At the phylum level (Supplementary Figure S2), the compositions of enriched microflora were different, but both were mostly dominated by Proteobacteria, Bacteroidetes, Firmicutes, and Actinobacteria. At the genus level (Additional File S4), the dominant components of the six cultured microflora were significantly (*p* < 0.05) different from each other. The largest single groups in each of the six microfloral cultures (R1, R2, R3, S1, S2, and S3, respectively) were *Myroides* (21.6%), *Sphingobacterium* (29.2%), *Acinetobacter* (59.1%), Unclassified genera (45.8%), *Orchrobactrum* (59.3%), and Unclassified genera (23.8%).

ANOVA results showed that the average relative abundance of unclassified genera was significantly (*p* < 0.05) higher in the S group than that in the R group (Supplementary Figure S3). Except for the unclassified genera, the average relative abundances of some genera, e.g., *Ochrobactrum*, *Massilia*, *Bacillus*, *Achromobacter*, *Comamonas*, *Pseudomonas*, were also higher in the enriched soil microflora, while the average relative abundances of other genera, e.g., *Acinetobacter*, *Enterobacter*, *Pedobacter*, *Myroides*, *Empedobacter*, *Stenotrophomonas*, *Sphingobacterium*, *Flavobacterium*, were higher in the cultured root microflora.

### The Soil Bacterial Community Shifted After Inoculation With Cultured Root and Soil Functional Microfloral Communities

**FIGURE 2:**
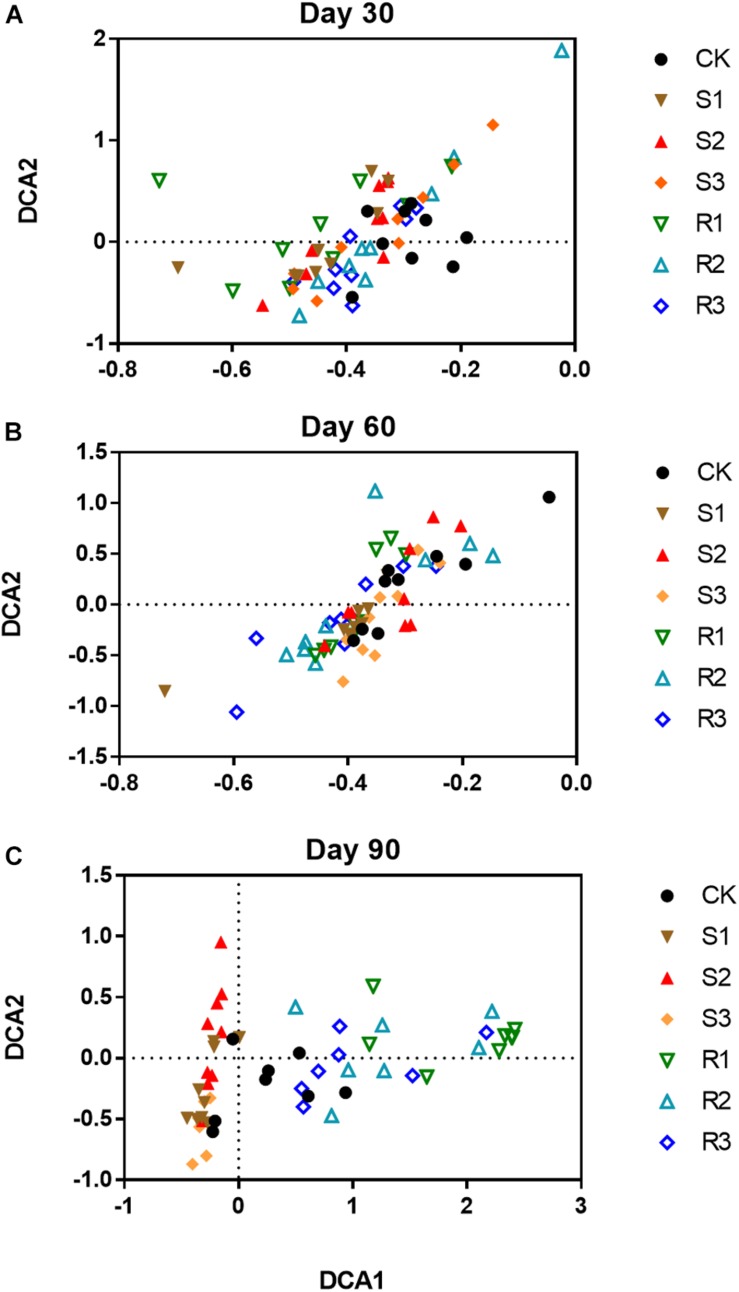
The ordination plots of all samples for the community structure analyzed by detrended correspondence analysis (DCA) on day 30 **(A)**, 60 **(B)**, and 90 **(C)**. CK: control group; Treatment with three root functional microflora (R1, R2, and R3) and with three soil functional microflora (S1, S2, and S3).

More than 30,000 high-quality 16S rRNA sequences were obtained for each sample. After clustering at 97% sequence identity, 10,262 OTUs were identified. DCA results (Supplementary Figure S4) showed that the bacterial community structure shifted over time (30, 60, and 90 days after plant inoculation) in each group. Although samples in different groups did not distinctly cluster together on day 30 (Figure 2A) or day 60 (Figure 2B), samples with root microfloral inoculation (R1, R2, and R3), soil microfloral inoculation (S1, S2, and S3), and the control group were relatively segregated well on day 90 (Figure 2C). Dissimilarity tests showed similar results that the functional microflora enriched from soil and root significantly (*p* < 0.05)influenced the soil bacterial community during plant growth (Table 1).

**TABLE 1:** Dissimilarity tests of bacterial community structure among treatments with different microflorae enriched from soil (S), root (R), and treatment with ddH_2_O (CK).

Groups	Time	MRPP^a^	ANOSIM^b^	ADONIS^c^
		**Delta**	**R**	**R^2^**
CK vs. S	Day 30	0.434*	0.06	0.053
	Day 60	0.463*	0.147	0.059*
	Day 90	0.442*	0.244*	0.104*
CK vs. R	Day 30	0.437*	0.148	0.068*
	Day 60	0.444	0.164	0.047
	Day 90	0.49*	0.352*	0.247*
S vs. R	Day 30	0.442	0.01	0.021
	Day 60	0.448*	0.082*	0.044*
	Day 90	0.477*	0.723*	0.417*

^*a*^Mutiple response permutation procedure, a nonparametric procedure that does not depend on assumptions such as normally distributed data or homogeneous variances, but rather depends on the internal variability of the data. ^*b*^Non-parametric multivariate analysis of variance (MANOVA) with the adonis function. ^*c*^Analysis of similarities. All three tests are non-parametric multivariate analyses based on dissimilarities among samples.The significant (p < 0.05) differences are indicated in bold and “*.”

OTUs were affiliated with 752 genera, and the soil bacterial communities were dominated (average relative abundance > 1%) by specific genera, including the unclassified genera (19.2–41.6%), *Gaiella* (0.05–8.9%), *Sphingomonas* (0.16–10.7%), *Gemmatimonas* (0.06–7.8%), *Cetobacterium* (0–17.7%), *Acidobacteria*_*Gp6* (0.04–6.4%), *Clostridium XlVb* (0–10.8%), *Acidobacteria*_*Gp4* (0.2–7.2%), *Acidobacteria*_*Gp16* (0.02–3.9%), *Nitrososphaera* (0.02–3.5%), *Acidobacteria*_*Gp3* (0.05–3.1%), *Solirubrobacter* (0.007–3.9%), *Clostridium XlVa* (0–7.8%), *Bacteroides* (0.005–7.3%), *Intrasporangium* (0.006–4.4%), *Nocardioides* (0.04–4.6%), *Arthrobacter* (0.03–3.9%), and *Acidobacteria*_*Gp7* (0.01–2.5%). The greatest difference in bacterial community structure was observed on day 90: 241 genera were significantly (*p* < 0.05) different between CK group and R group (Supplementary Table S2), 192 genera were significantly (*p* < 0.05) different between CK group and S group (Supplementary Table S3), and 388 genera were significantly (*p* < 0.05) different between S group and R group (Supplementary Table S4). The relative abundances of 122 genera in the R group, e.g., *Acinetobacter*, *Enterobacter*, *Comamonas*, *Cetobacterium*, *Bacteroides*, were significantly (*p* < 0.05) higher than those in the CK group, and the relative abundances of 154 genera in the S group, e.g., *Bacillus*, *Stenotrophomonas*, *Ochrobactrum*, *Achromobacter*, *Acinetobacter*, *Solirubarobacter*, and *Nitrososphaera*, were significantly (*p* < 0.05) higher than those in the CK group.

**FIGURE 3:**
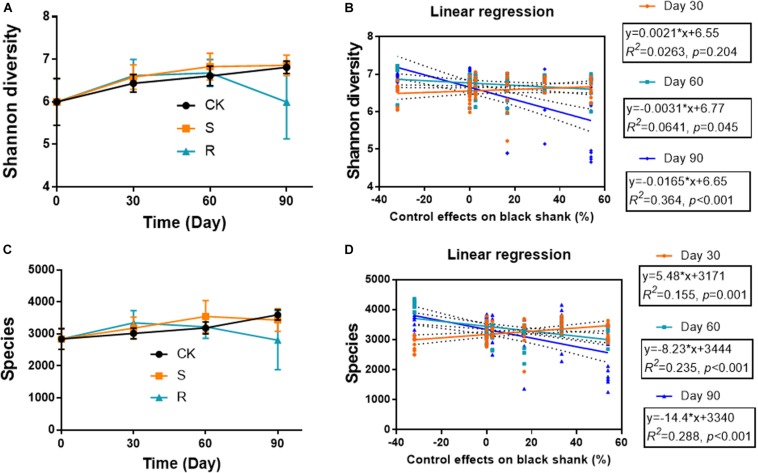
Temporal variations in **(A)** Shannon diversity and **(C)** species diversity; relationships between **(B)** Shannon diversity and suppression of black shank, and **(D)** species diversity and disease suppression **(D)**. CK, control group; R, treatment with root functional microflora; S, treatment with soil functional microflora.

The diversity indices (Shannon diversity and species diversity) varied during plant growth, and varied in different groups (Figures 3A,C). The microbial diversity increased continuously over time in the CK and S groups, while it also increased initially, but then decreased in the R group. On day 90, the Shannon diversity and species diversity were significantly (*p* < 0.05) lower in the R group (5.996 and 2805, respectively) compared with the CK group (6.812 and 3599) and the S group (6.857 and 3433). The indices showed no significant differences between CK and S groups. Linear regression analyses were conducted to explore the relationship between soil bacterial diversity and the suppression of black shank (Figures 3B,D). The results showed that the control effect was weakly but significantly positively correlated (*R*^2^ = 0.155, *p* = 0.001) with species diversity index on day 30, but it was weakly but significantly negatively correlated to the Shannon diversity on day 60 (*R*^2^ = 0.064, *p* = 0.045) and on day 90 (*R*^2^ = 0.364; *p* < 0.001). Similarly, weak but significantly negative correlations were found between control effect and species diversity on day 60 (*R*^2^ = 0.235, *p* < 0.001) and day 90 (*R*^2^ = 0.288; *p* < 0.001). These results suggested that some species of the cultured functional root or soil microflora could colonize the soil during crop growth, and they may play an important role in affecting the incidence of black shank.

### Network Interactions Shifted After Inoculation of Functional Microflora

**FIGURE 4:**
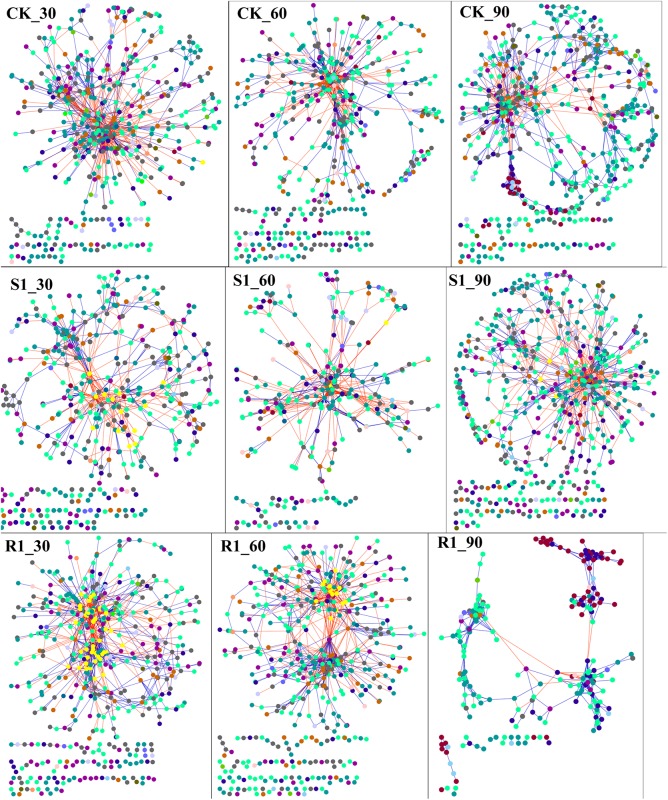
Shifts of molecular ecological networks in CK, S1, and R1 groups. The red line represented the negative correlation, and the blue line represented the positive correlation. The color of the circle represented the phylum of the node. CK, control group (*N* = 8); R1, treatment with functional microflora R1 (*N* = 8); S1, treatment with functional microflora S1 (*N* = 8).

In order to discern the ecological network structure of microbial communities, microbial population data was analyzed using the RMT-based network approach. Nine networks were constructed based on 16S rRNA gene sequencing data of S1 (with lowest disease suppression), R1 (with highest disease suppression), and CK group over the three planting time (day 30, 60, and 90), respectively (Figure 4). Major topological properties of the empirical MENs of microbial communities in the nine groups were shown in Supplementary Table S5. With a similar threshold (0.950∼0.990), their correlations were more than 0.600, indicating that the degree distributions in both the constructed molecular ecological networks fitted the power-law model well. If the same threshold (0.96) was considered, there were more nodes and links in the R1 group with higher disease suppression than those in CK group on day 30 (525 nodes, 1682 links in R1; 485 nodes, 1560 links in CK) and 60 (497 nodes, 1280 links in R1; 443 nodes, 1125 links in CK). Also, we found that there were the lowest nodes and links in the S1 group (442 nodes, 782 links) on day 30, which had the lowest disease suppression. Additionally, the positive links, the moduel, and modularity were increased with the inoculation of functional microflora.

### Isolation, Screening and Identification of Functional Strains

Seventy-four strains were obtained after isolation on a half-strength LB medium of 3 RFM and 3 SMF enrichments. Through antagonism experiments, 18 strains were found capable of inhibiting the growth of *P. nicotianae* HD1 (Supplementary Table S6), and Their 16S rDNA sequence analysis showed 12 strains had highest similarity to *Acinetobacter calcoaceticus* (99%), two strains to *Citrobacter amalonaticus* (99%), two strains to *Stenotrophomonas maltophilia* (99%), one strain to *Enterobacter cloacae* (99%), and one strain to *Bacillus* sp. (99%).

### Relationships Between Functional Strains/Genera and the Suppression During Plant Growth

Spearman’s ranked correlation tests were conducted at the genus and OTU levels to explore the relationship between the suppression of black shank and the above five isolated and potentially functional genera (Table 2). Among the 10,262 OTU counts of the 16S rRNA sequencing results for SUB5639076, nine OTUs were assigned to *Acinetobacter*, two assigned to *Enterobacter*, 16 assigned to *Bacillus*, two assigned to *Stenotrophomonas*, and zero assigned to *Citrobacter* (Table 2). Based on Spearman’s ranked correlation tests, *Acinetobacter* and *Enterobacter* were correlated with disease suppression at the genus level; at OTU level, suppression was significantly (*p* < 0.05) and positively correlated with relative abundances of two OTUs (OTU_2463, and OTU_2909) assigned to *Bacillus* on day 30, two OTUs (OTU_1196, and OTU_11902) assigned to *Bacillus* on day 60 and seven OTUs (four assigned to *Acinetobacter*, two assigned to *Enterobacter*, and one assigned to *Bacillus*) on day 90. Some significantly (*p* < 0.05) but negatively OTUs were also correlated with suppression, and it was interesting that the majority of OTUs showed the opposite correlation on the different planting stages.

**TABLE 2:** Correlated the control effects on black shank and functional OTUs/genera in soil bacterial communities during tobacco planting using Spearman’s ranked correlation tests.

Genus/OTU		Day 30		Day 60		Day 90	
			
		*r*	*p*	*r*	*p*	*r*	*p*
*Acinetobacter*	OTU_481	0.035	0.786	0.246	0.052	**−0.416**	**0.001**
	OTU_1936	−0.164	0.210	0.138	0.282	**0.587**	**0.000**
	OTU_1809	−0.092	0.485	0.083	0.516	0.143	0.303
	OTU_3797	**−0.495**	**0.000**	−	−	**0.772**	**0.000**
	OTU_1587	−	−	−	−	**0.621**	**0.000**
	OTU_3074	**−0.268**	**0.038**	−	−	−	−
	OTU_5227	−	−	−	−	**0.431**	**0.001**
	OTU_14667	−	−	−	−	−	−
	OTU_3184	−	−	−0.224	0.078	−	−
*Enterobacter*	OTU_1369	−0.088	0.505	**−0.390**	**0.002**	**0.499**	**0.000**
	OTU_6599	−0.028	0.832	−0.099	0.443	**0.662**	**0.000**
*Bacillus*	OTU_374	0.096	0.467	−0.013	0.919	**−0.304**	**0.026**
	OTU_1196	0.154	0.241	**0.315**	**0.012**	**−0.463**	**0.000**
	OTU_6478	0.236	0.069	−0.017	0.897	**0.385**	**0.004**
	OTU_549	−0.034	0.797	−0.127	0.322	**−0.508**	**0.000**
	OTU_1419	0.064	0.628	0.109	0.396	**−0.425**	**0.001**
	OTU_596	0.006	0.961	−0.059	0.649	**−0.434**	**0.001**
	OTU_2660	0.152	0.248	0.050	0.698	**−0.314**	**0.021**
	OTU_2463	**0.387**	**0.002**	0.002	0.990	0.257	0.061
	OTU_13019	0.254	0.050	−0.243	0.055	−0.220	0.110
	OTU_2825	0.093	0.482	−0.206	0.105	−0.131	0.345
	OTU_5995	0.242	0.062	0.112	0.383	−0.187	0.175
	OTU_8477	0.034	0.797	−0.147	0.251	−0.039	0.779
	OTU_2909	**0.261**	**0.044**	−0.019	0.884	0.008	0.955
	OTU_11902	0.195	0.136	**0.335**	**0.007**	−0.263	0.055
	OTU_2977	−0.100	0.445	−0.016	0.901	−	−
	OTU_7076	−	−	−	−	0.008	0.955
*Stenotrophomonas*	OTU_341	0.118	0.368	0.044	0.732	**−0.282**	**0.039**
	OTU_322	0.223	0.079	−0.160	0.211	**−0.290**	**0.033**
*Acinetobacter*		**−0.338**	**0.008**	**0.252**	**0.047**	**0.459**	**0.000**
*Enterobacter*		−0.065	0.620	**−0.326**	**0.009**	**0.635**	**0.000**
*Bacillus*		0.127	0.332	0.083	0.520	−0.255	0.063
*Stenotrophomonas*		0.110	0.403	−0.106	0.406	−0.259	0.059

**The significant (p < 0.05) correlations are indicated in bold.**

Random forest analysis was conducted with the relative abundance of 29 OTUs (on day 30, 60, and 90) to predict that this sample was from R, S, or CK group to evaluate the role and the relative importance of the above 29 functional OTUs in the field experiment. According to the results, the mean accuracy was 0.52, 0.46, and 0.85 on day 30, 60, and 90, respectively. Based on importance order (Supplementary Figure S5), the top five OTUs included OTU_481, OTU_1196, OTU_322, OTU_6478, and OTU_2660 on day 30, included OTU_1196, OTU_374, OTU_1369, OTU_549, and OTU_341 on day 60, and included OTU_3797, OTU_1196, OTU_481, OTU_1936, and OTU_341 on day 90.

### The Colonization and Variation of Functional Strains/Genera During Planting

**FIGURE 5:**
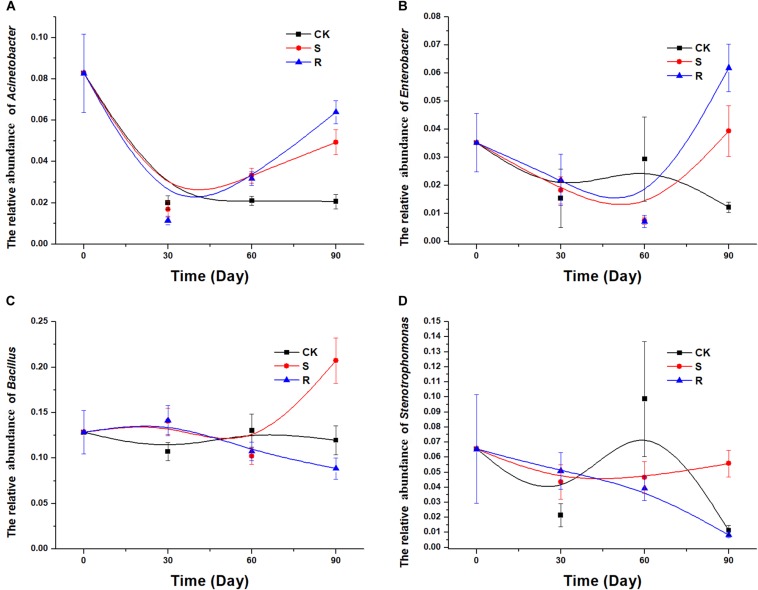
Relative abundance of several genera over time: **(A)** *Acinetobacter*, **(B)** *Enterobacter*, **(C)** *Bacillus*; **(D)** *Stenotrophomonas*. CK, control group (*N* = 8); *R*, treatment with root functional microflora (*N* = 24); S, treatment with soil functional microflora (*N* = 24).

After isolation and screening, 18 functional strains were identified to belong to five genera, including *Acinetobacter*, *Citrobacter*, *Stenotrophomonas*, *Enterobacter*, and *Bacillus*. Therefore, it was necessary to explore and compare the colonization/variation of these five genera with cultured RFM and SFM. In the 16S rRNA gene sequencing data of soil samples, *Citrobacter* was not detected. The population dynamics over time of the genera *Acinetobacter* (Figure 5A) and *Enterobacter* (Figure 5B) were similar. We observed a decrease for *Acinetobacter* and *Enterobacter* over time in the CK group, but compared with the continuous decrease of CK groups, their relative abundances decreased first and then increased in the groups with soil/root microfloral treatments during planting (Figures 5A,B). The population dynamics of the genera *Bacillus* (Figure 5C) and *Stenotrophomonas* (Figure 5D) were similar. In the CK group, the relative abundance of *Bacillus* and *Stenotrophomonas* decreased initially, then increased, and then decreased again. In the group with root microfloral treatments, the relative abundance of *Bacillus* and *Stenotrophomonas* continually decreased. And in the group with soil microfloral treatments, the relative abundance of *Bacillus* and *Stenotrophomonas* slowly increased.

We mapped the 16S rRNA gene sequences of the 18 strains onto the representative sequences of 29 functional OTUs. The results indicated that nine strains had the highest similarity to OTU_481 (>97%), and one strain had the highest similarity to OTU_322 (>97%). We further analyzed the dynamics of these two OTUs (Supplementary Figure S6) and found the relative abundance of the OTU_481 in the S group reached the highest on day 30, and in the R and CK group on day 60. Differently, the relative abundance of OTU_322 in the R group achieved the highest on day 30, and in the S and CK group on day 60.

**FIGURE 6:**
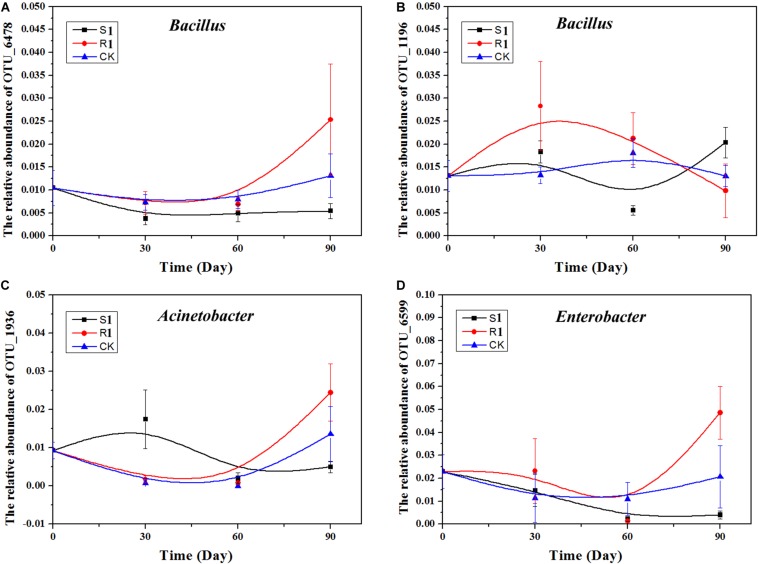
Relative abundance of four OTUs over time: **(A)** *Acinetobacter*, **(B)** *Enterobacter*, **(C)** *Bacillus*; **(D)** *Stenotrophomonas*. CK, control group (*N* = 8); R1, treatment with functional microflora R1 (*N* = 8); S1, treatment with functional microflora S1 (*N* = 8).

Based on the above nine networks analysis, OTU_6478 (*Bacillus*), OTU_1196 (*Bacillus*), OTU_6599 (*Enterobacter*), and OTU_1936 (*Acinetobacter*), which were the top 10 important OTUs in the random forest analysis, were possible key functional OTUs. Moreover, the relative abundances of these four OTUs were also significantly (*p* < 0.05) and positively correlated with the suppression of black shank. Therefore, we further analyzed the dynamics of these four OTUs (Figure 6). Compared with the S1 and the control groups, the relative abundances of these four OTUs varied greater in the R1 group. In the R1 group, the relative abundances of OTU_6478, OTU_6599, and OTU_1936 achieved the highest on day 90, while the relative abundances of OTU_1196 reached the highest on day 30.

## Discussion

Although the bacterial community structure and composition are different in plant tissues vs. soil environments (Thokchom et al., 2017), similar functional species can be observed or isolated (Falcao et al., 2014; You et al., 2016). However, previous studies have mainly focused on the relationships between soil bacterial community compositions and the incidence of crop diseases (Mengesha et al., 2017). It is not sure which is more effective for plant disease control, microflora in plant tissues, or from the soil environment. We found that RFM had a greater effect on the diversity, composition, and structure of soil bacterial communities during plant growth, and were also more effective in controlling black shank compared to SFM, and RFM not only inoculated more functional strains, which may be more successful in colonizing on plant roots or soil environment, but also strengthened the interaction among soil microbial communities.

Functional strains play important roles in protecting plants from pathogens (Weyens et al., 2009). One reason is that their metabolites can effectively inhibit the growth of pathogens (Ma et al., 2018). Recently, many biocontrol agents had been isolated from plant tissues and soil to control plant diseases in recent years (Falcao et al., 2014; You et al., 2016), indicating that both endophytes and surrounding soil microorganisms of plants could play important roles in disease resistance. In this study, we found that more functional microfloral communities were from plant roots than from the rhizosphere soil, suggesting that root endophytes could play more important roles in controlling the disease. Also, we isolated more culturable functional strains from RFM than from SFM, suggesting more functional strains in plant roots. More functional strains provide the basic conditions for better control of plant pathogens.

Whereafter, microbial colonization may determine whether the functional strains can play their roles in suppressing the incidence of black shank during plant growth (Compant et al., 2010). It is necessary to minimize niche vacancy and effectively fill vacant niches before colonizing (Benbow and Sugar, 1999; Hallmann et al., 2001; Sang and Kim, 2014). Root endophytes are substantially different from soil microbial communities, so that their niches should be more complementary. Our results indicated that the inoculations of RFM or SFM cultures altered the diversity, composition, and structure of the soil bacterial communities, suggesting that colonization had occurred. We further observed that the relative abundance of functional genera or strains (e.g., OTU_481 and OTU_322) increased on different planting periods after the inoculation of SFM and RFM in comparison to the control group, further suggesting such colonization effects. However, greater effects of RFM were reflected in the larger number of genera (241) than SFM (192), which were both significantly different from the control group. Speculatively, there might be more microbes of RFM than SFM colonizing soil and replacing some indigenous microbes, resulting in the disappearance of rare species.

Another reason for more species colonization after RMF inoculation is that root endophytes might be more physically proximal to plants compared to soil microbes. Soil microbes may work together in the soil-microbe-plant systems to mediate and influence various nutrient exchanges and microenvironments (Chaparro et al., 2012). For example, the surrounding soil microbes were influenced by both plant root exudates and soil properties, such as pH, temperature, humidity, and air (Hu et al., 2018). In turn, adding beneficial soil microorganisms to those already present in the soil could improve the diversity, composition and structure of soil microbial communities to optimize soil health, maximize plant nutrient uptake, increase plant growth, confer resistance to abiotic stresses and suppress diseases (Chaparro et al., 2012). However, soil microbes are highly variable and are susceptible to changes in the external natural environment and to human factors, causing unstable functions. Although root endophytes are also influenced by natural environmental and social factors, they are mainly dominated by the plant internal environment and growth needs. Recent research has focused on plant endophytes and their potential applications, such as increasing yield (Sturz et al., 2010; Rashid et al., 2012; Falcao et al., 2014), phytoremediation of organic pollutants (Afzal et al., 2014), or heavy metals (Luo et al., 2011), disease resistance (Berg and Hallmann, 2006), and stress tolerance (Miliute et al., 2015). Therefore, root endophytes, rather than soil microbes, might be more effective in agricultural applications.

Besides, it might be other reason for stronger disease suppression in RFM. For example, RFM inoculation may cause more closely interactions among microorganisms. A previous study indicated that the close interactions among microorganisms were decreased, meaning that the ability of the plant to resist the pathogen was weakened, resulting in high morbidity and yield loss (Xiao et al., 2018). On the contrary, more closely interactions may strengthen the function of the microbial communities. In this study, we found more positive interactions with RFM inoculations, indicating that synergies between microbes were enhanced.

In our study, 18 functional strains associated with genera *Acinetobacter* (*A. calcoaceticus*), *Enterobacter* (*E. cloacae*), *Bacillus*, *Stenotrophomonas* (*S. maltophilia*), and *Citrobacter* (*C. amalonaticus*) showed suppressive effects on the growth of the pathogen *P. nicotianae*. In many previous studies, some strains of these five genera were able to suppress or inhibit plant pathogens. Liu et al. (2007) found that an *Acinetobacter* strain, *A. baumannii* LCH001, showed strong growth inhibition against several phytopathogens, including *Phytophthora capsici*, *Fusarium graminearum*, and *Rhizoctonia solani*. Utkhede and Sholberg (1986) found *E. aerogenes* was antagonistic to 12 kinds of plant pathogens. In the study of Dunne et al. (1997), *S. maltophilia* W81 could control the growth of the pathogen *Pythium ultimum* mediated by extracellular proteolytic activity. One compound isolated from *C. freundii* exhibited antimicrobial activity against a wide range of Gram-negative bacteria (Shanks et al., 2012). Han et al. (2016) reported that the effect of *B. subtilis* Tpb55 on controlling black shank was correlated with the ability to inhibit mycelial growth and ability to successfully colonize the plant roots. In this study, although *Citrobacter* was not detected in the soil during plant growth, four other functional genera were detected (Figure 5). Spearman’s ranked correlation tests, and random forest analysis showed that some strains of *Bacillus* might control the incidence of black shank at an early stage, while *Acinetobacter*, *Stenotrophomonas*, and *Enterobacter* might play roles at a later stage. We conclude that some strains of *Acinetobacter*, *Stenotrophomonas*, *Enterobacter*, and *Bacillus* may be potential agents in the biocontrol of black shank.

The mechanism of microbial inhibition of plant diseases (Mendes et al., 2013; Hacquard et al., 2017) and human/animal diseases (Barratt, 2000; Sanders et al., 2013) share similarities in that they are mainly via the antagonistic action of microorganisms on pathogen growth. Recent research reported that microbiota transplantation had an enormous potential to prevent and cure human/animal diseases like ulcerative colitis (Borody and Khoruts, 2012; Smits et al., 2013). Similarly, although most research has been focused on the application of single biocontrol agents in controlling plant disease (Thokchom et al., 2017; Ma et al., 2018), microbiota transplantation may have more enormous potentials. Because intestinal microbiota alters with age, microbiota transplantation therapy is different in adults vs. children (Sha et al., 2014). For plants, the microbial communities are different at different planting stages (Xiao et al., 2018), so that transplantation stages of diverse microbiota should also be considered in the biocontrol process. Another aspect is the appropriate frequency of microbiota transplantation, such as once per day, once per two days, or even once per 30 days. Long-term consumption of probiotics has been reported to be beneficial to improve intestinal microbiota (Almeida et al., 2012). However, many of microbiota also have short-term side effects, such as a loss of effectiveness with extended use, and long-term effects have yet to be quantified (Click, 2014). Therefore, future research should examine when and how microbiota transplantation may be most effectively applied in biocontrol of plant diseases. Furthermore, studies are needed to understand whether microbial communities obtained from plant interiors or exteriors are more efficient in the reduction of diseases than single targeted biocontrol organisms, and to better examine the interactions between these organisms and their potential diverse roles and division of labor in combatting diseases.

## Conclusion

In this study, we found that the functional microflora from plant roots might be more effective in controlling black shank than those from the soil. RFM not only inoculated more functional strains, which might be successful in colonizing, but also strengthened the interaction among soil microbial communities. OTUs assigned to *Acinetobacter*, *Enterobacter*, *Bacillus*, *Stenotrophomonas*, or individual microflora may be developed for biocontrol of black shank.

## Data Availability Statement

The data can be found in NCBI under accession numbers PRJNA543688 and PRJNA543952.

## Ethics Statement

This article does not contain any studies with human participants or animals performed by any of the authors. All plant procedures were approved by the Tobacco Research Institute, Chinese Academy of Agricultural Sciences.

## Author Contributions

WC and QT conceived the experiment. TL, ZZ, and JY performed the experiment. TL and YX analyzed the data and wrote drafts of the manuscript. TH and TY revised and further edited the manuscript. All authors have read and approved the manuscript.

## Conflict of Interest

The authors declare that the research was conducted in the absence of any commercial or financial relationships that could be construed as a potential conflict of interest.

## Abbreviations

RMF: Root functional microflora
SMF: Soil functional microflora
OUT: Operational taxonomic unit
DP_S: Soil of disease plant
DP_R: Root of disease plant
HP_S: Soil of healthy plant
HP_R: Root of healthy plant
ANOVA: A one-way analysis of variance
DCA: Detrended correspondence analysis.
