# Supplementary material for: Effects of Cultured Root and Soil Microbial Communities on the Disease of Nicotiana tabacum Caused by Phytophthora nicotianae
Source: Front Microbiol. 2020 May 15;11:929. doi: 10.3389/fmicb.2020.00929 (PMC7243367; doi:10.3389/fmicb.2020.00929)
Supplement: Supplementary file 1 [file Data_Sheet_1.PDF]

Table S1 Results of dual culture antagonistism experiments on pathogen *Phytophthora nicotianae* HD1

Rhizosphere soil microflora				Root endophytic microflorae			
Diseased plants		Healthy plant		Diseased plants		Healthy plant	
Sample ID	Inhibition zone	Sample ID	Inhibition zone	Sample ID	Inhibition zone	Sample ID	Inhibition zone
DP_S1	+	HP_S1	+	DP_R1	+	HP_R1	-
DP_S2	-	HP_S2	-	DP_R2	-	HP_R2	+
DP_S3	-	HP_S3	+	DP_R3	+	HP_R3	+
DP_S4	-	HP_S4	+	DP_R4	-	HP_R4	+
DP_S5	+	HP_S5	-	DP_R5	+	HP_R5	+
DP_S6	+	HP_S6	-	DP_R6	+	HP_R6	+
DP_S7	-	HP_S7	-	DP_R7	-	HP_R7	+
DP_S8	-	HP_S8	+	DP_R8	-	HP_R8	+
DP_S9	+	HP_S9	-	DP_R9	+	HP_R9	+
DP_S10	-	HP_S10	+	DP_R10	+	HP_R10	+
DP_S11	-	HP_S11	-	DP_R11	+	HP_R11	+
DP_S12	+	HP_S12	+	DP_R12	+	HP_R12	+
DP_S13	+	HP_S13	-	DP_R13	+	HP_R13	-
DP_S14	+	HP_S14	-	DP_R14	+	HP_R14	-
DP_S15	-	HP_S15	-	DP_R15	+	HP_R15	+
DP_S16	+	HP_S16	+	DP_R16	+	HP_R16	+
DP_S17	+	HP_S17	+	DP_R17	-	HP_R17	+
DP_S18	-	HP_S18	-	DP_R18	+	HP_R18	+
DP_S19	-	HP_S19	-	DP_R19	+	HP_R19	+
DP_S20	+	HP_S20	-	DP_R20	+	HP_R20	-

"+" indicates that the microflora could inhibit the growth of *P. nicotianae* HD1, and "-" indicates that the microflora could not.
