# Supplementary material for: Effects of Cultured Root and Soil Microbial Communities on the Disease of Nicotiana tabacum Caused by Phytophthora nicotianae
Source: Front Microbiol. 2020 May 15;11:929. doi: 10.3389/fmicb.2020.00929 (PMC7243367; doi:10.3389/fmicb.2020.00929)
Supplement: Supplementary file 2 [file Data_Sheet_2.PDF]

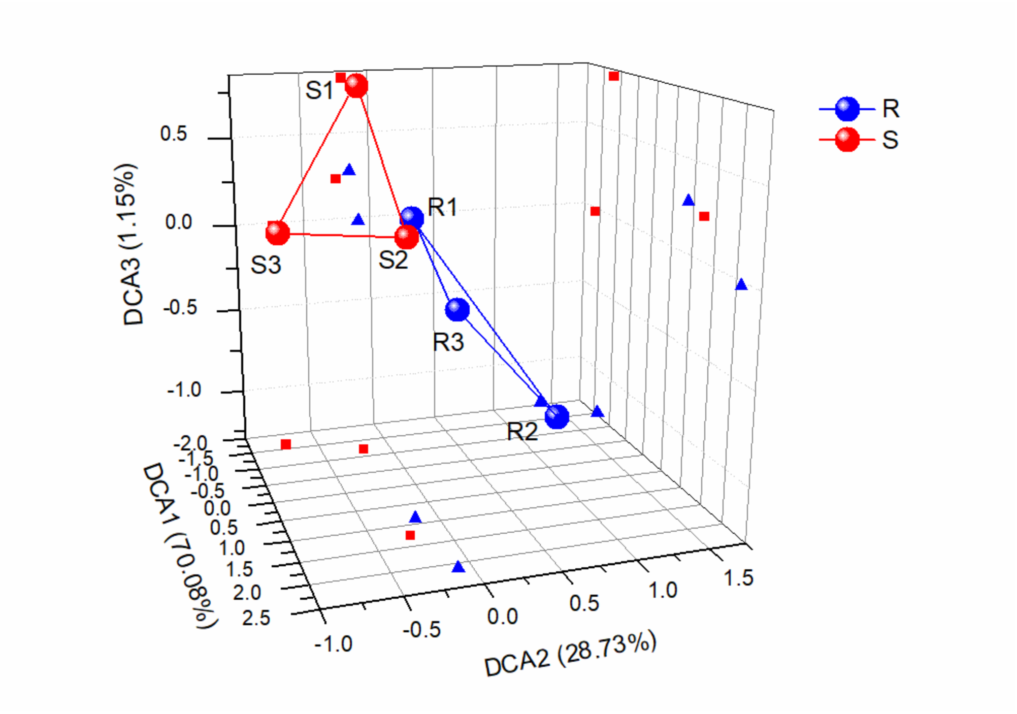

Fig. S1 The ordination plots of the selected 3 root functional microflorae (R: R1, R2, and R3) and 3 soil functional microflorae (S: S1, S2, and S3) for the community structure analyzed by detrended correspondence analysi
