# Supplementary material for: Effects of Cultured Root and Soil Microbial Communities on the Disease of Nicotiana tabacum Caused by Phytophthora nicotianae
Source: Front Microbiol. 2020 May 15;11:929. doi: 10.3389/fmicb.2020.00929 (PMC7243367; doi:10.3389/fmicb.2020.00929)
Supplement: Supplementary file 5 [file Data_Sheet_5.PDF]

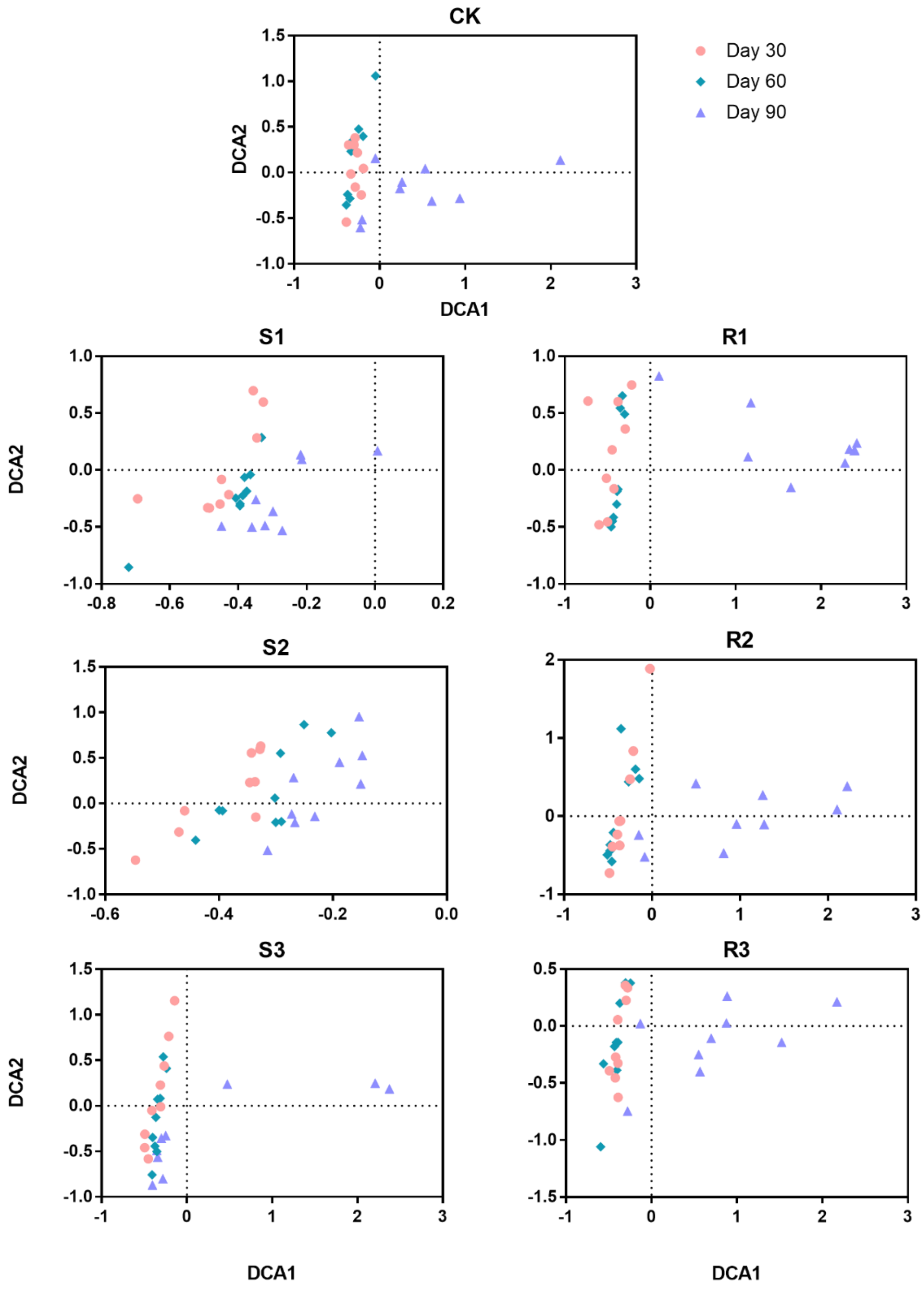

Fig. S4 The ordination plots of all samples for the community structure analyzed by detrended correspondence analysis (DCA) in different treatments. CK: control group; Treatment with three root functional microflorae (R group: R1, R2 and R3) and with three soil functional microflorae (S group: S1, S2 and S3).
