# Supplementary material for: Effects of Cultured Root and Soil Microbial Communities on the Disease of Nicotiana tabacum Caused by Phytophthora nicotianae
Source: Front Microbiol. 2020 May 15;11:929. doi: 10.3389/fmicb.2020.00929 (PMC7243367; doi:10.3389/fmicb.2020.00929)
Supplement: Supplementary file 6 [file Data_Sheet_6.pdf]

Table S2 Significantly (p-value < 0.05) different genera in R group and CK group					
Genus	CK: mean rel. freq. (%)	CK: std. dev. (%)	R: mean rel. freq. (%)	R: std. dev. (%)	p-values
Cetobacterium	1.604	1.339	6.815	4.362	0
Clostridium XlVb	0.608	0.579	5.213	3.199	0
Unclassified	27.675	2.796	32.144	4.915	0.007
Clostridium XlVa	0.723	0.677	2.831	1.863	0
Bacteroides	0.631	0.476	2.497	1.535	0
Barnesiella	0.455	0.464	1.934	1.347	0
Bifidobacterium	0.177	0.129	0.833	0.475	0
Lactobacillus	0.207	0.193	0.809	0.488	0
Peptococcus	0.165	0.148	0.682	0.449	0
Parabacteroides	0.173	0.151	0.682	0.436	0
Roseburia	0.269	0.281	0.7	0.408	0.006
Aeromonas	0.107	0.087	0.503	0.388	0
Turicibacter	0.07	0.063	0.456	0.304	0
Clostridium sensu stricto	0.186	0.202	0.564	0.32	0.002
Dysgonomonas	0.104	0.09	0.455	0.258	0
Lachnospiracea_incertae_sedis	0.144	0.192	0.441	0.323	0.008
Pseudoflavonifractor	0.105	0.104	0.39	0.269	0
Anaerorhabdus	0.057	0.046	0.33	0.316	0.001
Lactovum	0.082	0.066	0.316	0.165	0
Ruminococcus	0.041	0.033	0.191	0.116	0
Blautia	0.046	0.031	0.196	0.159	0
Robinsoniella	0.042	0.038	0.183	0.141	0
Anaerotruncus	0.06	0.058	0.194	0.135	0.001
Stomatobaculum	0.081	0.099	0.213	0.134	0.013
Flavonifractor	0.046	0.042	0.161	0.104	0
Intestinimonas	0.034	0.03	0.14	0.121	0.001
Clostridium IV	0.062	0.051	0.157	0.092	0.003
Acetatifactor	0.013	0.014	0.1	0.081	0
Eubacterium	0.035	0.032	0.114	0.073	0.001
Clostridium XVIII	0.024	0.023	0.102	0.079	0
Buttiauxella	0.023	0.016	0.097	0.065	0
Euzebya	0.026	0.037	0.1	0.148	0.044
Anaerostipes	0.01	0.008	0.079	0.059	0
Romboutsia	0.019	0.012	0.087	0.057	0
Marvinbryantia	0.02	0.018	0.087	0.066	0
Shewanella	0.044	0.043	0.11	0.068	0.007
Escherichia/Shigella	0.016	0.01	0.07	0.099	0.021
Hydrogenoanaerobacterium	0.014	0.014	0.064	0.053	0.001
Dorea	0.022	0.017	0.071	0.05	0.001
Enterobacter	0.012	0.005	0.062	0.038	0
Fusicatenibacter	0.007	0.008	0.054	0.056	0.001
Acinetobacter	0.021	0.01	0.064	0.026	0
Ruminococcus2	0.011	0.009	0.042	0.048	0.009
GpI	0.012	0.009	0.04	0.058	0.041
Butyricicoccus	0.006	0.005	0.034	0.024	0
Fusobacterium	0.006	0.004	0.033	0.055	0.035
Mangrovibacterium	0.01	0.011	0.033	0.022	0.001
Pseudorhodoferax	0.005	0.004	0.028	0.013	0
Intestinibacter	0.015	0.011	0.037	0.024	0.003

Allobaculum	0.004	0.004	0.023	0.023	0.002
Veillonella	0.002	0.002	0.02	0.026	0.006
ysipelotrichaceae_incertae_sed	0.004	0.005	0.021	0.018	0.001
Neisseria	0.002	0.002	0.018	0.033	0.029
Enterorhabdus	0.004	0.005	0.02	0.018	0.001
Anaerovorax	0.004	0.005	0.018	0.016	0.001
Porphyromonas	0.001	0.002	0.015	0.025	0.022
Paracoccus	0.006	0.008	0.02	0.019	0.014
Akkermansia	0.002	0.003	0.015	0.012	0
Rhodomicrobium	0.003	0.004	0.015	0.022	0.03
Phyllobacterium	0.004	0.002	0.015	0.013	0.001
Haemophilus	0.001	0.002	0.012	0.016	0.008
Erythrobacter	0.004	0.003	0.014	0.018	0.021
Mucispirillum	0.002	0.002	0.012	0.009	0
Comamonas	0.004	0.004	0.013	0.015	0.023
Staphylococcus	0.001	0.001	0.008	0.006	0
Coprobacillus	0.002	0.003	0.009	0.006	0
Deinococcus	0	0	0.005	0.008	0.009
Vibrio	0.002	0.002	0.007	0.005	0.002
Dialister	0.002	0.002	0.006	0.005	0.006
Actinotalea	0.003	0.002	0.007	0.008	0.037
Rubricoccus	0	0	0.004	0.006	0.002
Collinsella	0	0	0.004	0.007	0.009
Cloacibacterium	0.001	0.002	0.005	0.005	0.011
GpIIa	0	0	0.004	0.005	0.003
Helicobacter	0.002	0.002	0.006	0.004	0.005
Plesiomonas	0.001	0.001	0.004	0.004	0
Exiguobacterium	0	0	0.003	0.003	0
Psychrilyobacter	0	0	0.003	0.005	0.01
Lachnospira	0.002	0.002	0.005	0.005	0.019
Nitriliruptor	0	0	0.003	0.006	0.039
Megasphaera	0	0	0.003	0.003	0
Streptobacillus	0	0	0.003	0.004	0.005
Spirosoma	0	0	0.003	0.005	0.024
Treponema	0	0	0.002	0.003	0
Delftia	0.001	0.001	0.003	0.004	0.016
Luteibacter	0	0	0.002	0.005	0.034
Planomicrobium	0	0	0.002	0.004	0.017
Paraprevotella	0	0	0.002	0.003	0.002
Candidatus Pelagibacter	0	0	0.002	0.003	0.003
Acidovorax	0.002	0.001	0.004	0.004	0.046
Odoribacter	0	0	0.002	0.003	0.006
Mycoplasma	0	0	0.002	0.003	0.003
Weissella	0	0	0.002	0.003	0.017
Desemzia	0	0	0.002	0.002	0
Filifactor	0	0	0.002	0.003	0.005
Filomicrobium	0	0	0.002	0.002	0.001
Thauera	0	0	0.002	0.003	0.012
Clostridium III	0	0.001	0.002	0.003	0.025
Asaia	0	0	0.002	0.002	0.001
Psychrobacter	0	0	0.001	0.002	0.005
Lactonifactor	0	0.001	0.002	0.002	0.03
Rhodoferax	0	0	0.001	0.002	0.013
Hansschlegelia	0	0	0.001	0.003	0.024

Dechloromonas	0	0	0.001	0.003	0.033
Nitrobacter	0	0	0.001	0.002	0.005
Peptoniphilus	0	0	0.001	0.002	0.029
Desulfovibrio	0	0	0.001	0.002	0.008
Lachnoanaerobaculum	0	0	0.001	0.002	0.01
Selenomonas	0	0	0.001	0.002	0.025
Atopobium	0	0	0.001	0.002	0.046
Jiangella	0	0	0.001	0.002	0.037
Senegalimassilia	0	0	0.001	0.002	0.03
Finegoldia	0	0	0.001	0.002	0.024
Lentzea	0	0	0.001	0.001	0.005
Fretibacterium	0	0	0.001	0.001	0.017
Permianibacter	0	0	0.001	0.001	0.026
Solibacillus	0	0	0.001	0.001	0.016
Ammoniphilus	0	0	0.001	0.001	0.031
WPS-2_genera_incertae_sedis	0	0	0	0.001	0.031
Parvibaculum	0	0	0	0.001	0.029
Inhella	0	0	0	0.001	0.029
Desulfomonile	0	0	0	0.001	0.043
Albidovulum	0.002	0.001	0	0	0.023
Hydrogenispora	0.002	0.001	0	0.001	0.002
Pyxidicoccus	0.006	0.002	0.004	0.004	0.049
Rudaea	0.003	0.002	0.001	0.001	0.016
Haliscomenobacter	0.004	0.003	0.001	0.002	0.042
Lysinibacillus	0.003	0.003	0	0.001	0.03
Solimonas	0.006	0.003	0.002	0.004	0.029
Ardenticatena	0.004	0.002	0.001	0.002	0.003
Aquisphaera	0.004	0.003	0.001	0.002	0.024
Pirellula	0.004	0.004	0	0.001	0.037
Methanomassiliicoccus	0.009	0.004	0.004	0.005	0.032
Vulcaniibacterium	0.005	0.003	0	0	0.003
Terrimicrobium	0.008	0.005	0.002	0.003	0.024
Nitrospirillum	0.007	0.004	0.002	0.003	0.007
Diplorickettsia	0.006	0.003	0	0	0.003
BRC1_genera_incertae_sedis	0.009	0.007	0.002	0.003	0.04
Smaragdicoccus	0.01	0.007	0.002	0.003	0.031
Pilimelia	0.01	0.005	0.002	0.003	0.005
Tepidisphaera	0.011	0.008	0.003	0.004	0.027
Gemmata	0.011	0.004	0.003	0.004	0.001
Paludibaculum	0.009	0.008	0.001	0.002	0.03
Gp15	0.014	0.009	0.004	0.007	0.024
Rhizobacter	0.022	0.005	0.01	0.009	0
Desulfocarbo	0.016	0.012	0.004	0.008	0.038
Legionella	0.021	0.011	0.009	0.008	0.021
Brevibacillus	0.015	0.012	0.002	0.003	0.031
Sediminibacterium	0.022	0.006	0.009	0.013	0.001
Umezawaea	0.025	0.015	0.011	0.012	0.04
Blastochloris	0.021	0.013	0.006	0.007	0.021
Zavarzinella	0.019	0.008	0.003	0.004	0.001
Pelomonas	0.024	0.016	0.009	0.009	0.041
Bdellovibrio	0.039	0.013	0.022	0.021	0.022
Alsobacter	0.031	0.009	0.012	0.012	0.001
Parafilimonas	0.028	0.014	0.01	0.01	0.011
Chloroflexus	0.033	0.02	0.013	0.014	0.031

Piscinibacter	0.033	0.016	0.011	0.013	0.008
Bauldia	0.041	0.018	0.019	0.024	0.021
Microlunatus	0.059	0.017	0.036	0.037	0.034
Aggregicoccus	0.038	0.007	0.015	0.015	0
Labrys	0.036	0.016	0.012	0.013	0.005
Longilinea	0.04	0.022	0.015	0.015	0.024
Gp25	0.042	0.013	0.017	0.023	0.002
Aquabacterium	0.04	0.026	0.012	0.014	0.028
Catelliglobosispora	0.045	0.021	0.017	0.022	0.01
Novosphingobium	0.072	0.029	0.042	0.036	0.039
Bacillus	0.12	0.016	0.089	0.012	0.048
Aquicella	0.053	0.033	0.022	0.021	0.041
Hyphomicrobium	0.062	0.029	0.028	0.022	0.018
Phaselicystis	0.093	0.027	0.059	0.047	0.026
Allokutzneria	0.044	0.021	0.008	0.01	0.002
Nitrosospira	0.111	0.025	0.075	0.065	0.044
Herpetosiphon	0.053	0.033	0.016	0.016	0.021
Kitasatospora	0.06	0.025	0.022	0.016	0.003
Sporichthya	0.056	0.038	0.018	0.015	0.03
Haliangium	0.08	0.036	0.039	0.044	0.028
Gp18	0.061	0.018	0.02	0.028	0
Cupriavidus	0.051	0.04	0.009	0.011	0.028
Amycolatopsis	0.073	0.026	0.031	0.042	0.005
Rhodoligotrophos	0.084	0.022	0.04	0.033	0.001
Bosea	0.074	0.037	0.029	0.022	0.015
Labilithrix	0.094	0.037	0.043	0.033	0.007
Methyloceanibacter	0.096	0.039	0.038	0.04	0.006
Armatimonadetes_gp5	0.086	0.026	0.024	0.025	0
Kribbella	0.147	0.04	0.084	0.072	0.008
Mesorhizobium	0.159	0.044	0.094	0.094	0.021
Caldilinea	0.096	0.044	0.031	0.027	0.005
Armatimonadetes_gp4	0.095	0.026	0.028	0.027	0
Gp5	0.165	0.045	0.094	0.115	0.026
Anaeromyxobacter	0.138	0.082	0.06	0.072	0.045
WPS-1_genera_incertae_sedis	0.115	0.039	0.033	0.026	0.001
Blastocatella	0.172	0.049	0.081	0.056	0.001
Gp11	0.129	0.086	0.038	0.05	0.027
Niastella	0.132	0.061	0.038	0.034	0.004
Pseudoduganella	0.138	0.049	0.036	0.041	0.001
Aeromicrobium	0.216	0.046	0.112	0.087	0.001
Methyloversatilis	0.182	0.038	0.077	0.061	0
Povalibacter	0.216	0.075	0.102	0.088	0.005
Arenimonas	0.193	0.063	0.075	0.069	0.001
Aridibacter	0.277	0.066	0.157	0.122	0.003
Phenylobacterium	0.269	0.071	0.144	0.114	0.003
Lechevalieria	0.18	0.078	0.053	0.048	0.003
Conexibacter	0.385	0.087	0.255	0.166	0.014
Pedomicrobium	0.227	0.07	0.095	0.125	0.002
Streptomyces	0.332	0.146	0.164	0.129	0.02
Lacibacterium	0.326	0.157	0.15	0.159	0.025
hthonomonas/Armatimonadetes_gp	0.226	0.108	0.047	0.047	0.003
Chitinophaga	0.355	0.133	0.174	0.173	0.011
Mycobacterium	0.369	0.15	0.177	0.146	0.013
Gp17	0.323	0.177	0.125	0.162	0.024

Phycicoccus	0.315	0.121	0.114	0.103	0.003
Ramlibacter	0.345	0.126	0.137	0.146	0.003
Iamia	0.413	0.183	0.201	0.149	0.019
Terrimonas	0.347	0.195	0.133	0.133	0.024
Bradyrhizobium	0.486	0.135	0.261	0.233	0.005
escibacteria_genera_incertae_se	0.408	0.163	0.156	0.237	0.006
Agromyces	0.411	0.231	0.158	0.16	0.024
Gp10	0.376	0.148	0.119	0.134	0.002
Rhodoplanes	0.502	0.184	0.195	0.189	0.003
ıbdivision3_genera_incertae_sed	0.561	0.26	0.214	0.198	0.009
Aquihabitans	0.723	0.15	0.362	0.253	0
Marmoricola	0.696	0.175	0.323	0.219	0
Thermoleophilum	0.689	0.312	0.306	0.247	0.014
ırtobacteria_genera_incertae_se	0.78	0.293	0.331	0.327	0.005
Gp7	0.951	0.175	0.451	0.413	0
Nitrospira	0.874	0.212	0.359	0.311	0
Lysobacter	0.767	0.391	0.23	0.259	0.008
Solirubrobacter	0.968	0.276	0.412	0.288	0.001
Intrasporangium	1.169	0.439	0.602	0.824	0.03
Kofleria	1.043	0.344	0.452	0.391	0.002
Aciditerrimonas	1.027	0.303	0.429	0.284	0.001
Ilumatobacter	1.036	0.293	0.388	0.259	0
Gp3	1.605	0.314	0.835	0.621	0
Nocardioides	1.352	0.628	0.564	0.4	0.013
Arthrobacter	1.744	1.066	0.569	0.396	0.022
Gp16	2.516	0.666	0.929	0.735	0
Gp4	2.67	0.55	0.954	1.027	0
Gp6	3.292	0.979	1.454	1.202	0.001
Sphingomonas	4.421	1.786	2.352	1.79	0.021
Gemmatimonas	4.803	1.123	2.223	1.664	0
Gaiella	5.653	1.807	2.447	2.032	0.002
