# Supplementary material for: Effects of Cultured Root and Soil Microbial Communities on the Disease of Nicotiana tabacum Caused by Phytophthora nicotianae
Source: Front Microbiol. 2020 May 15;11:929. doi: 10.3389/fmicb.2020.00929 (PMC7243367; doi:10.3389/fmicb.2020.00929)
Supplement: Supplementary file 7 [file Data_Sheet_7.pdf]

Table S3 Significantly (p-value < 0.05) different genera in S group and CK group					
Genus	CK: mean rel. freq. (%)	CK: std. dev. (%)	S: mean rel. freq. (%)	S: std. dev. (%)	p-values
Solirubrobacter	0.968	0.276	2.209	0.821	0
Nitrososphaera	1.353	0.255	2.029	0.887	0.003
Gp7	0.951	0.175	1.553	0.541	0
Chitinophaga	0.355	0.133	0.704	0.343	0
Rhodoplanes	0.502	0.184	0.731	0.277	0.021
Mycobacterium	0.369	0.15	0.561	0.297	0.032
Gp10	0.376	0.148	0.564	0.238	0.021
Saccharibacteria_genera_incertae_sedis	0.238	0.066	0.41	0.196	0.001
Pseudolabrys	0.117	0.035	0.269	0.158	0
Microbacterium	0.084	0.035	0.22	0.267	0.024
Mesorhizobium	0.159	0.044	0.285	0.183	0.005
Gp5	0.165	0.045	0.29	0.143	0.001
Ktedonobacter	0.065	0.025	0.185	0.249	0.032
Reyranella	0.206	0.028	0.318	0.123	0
Lechevalieria	0.18	0.078	0.286	0.153	0.023
Conexibacter	0.385	0.087	0.487	0.149	0.035
Streptophyta	0.016	0.013	0.115	0.061	0
Bacillus	0.12	0.046	0.207	0.119	0.007
Microlunatus	0.059	0.017	0.144	0.063	0
Ensifer	0.083	0.049	0.157	0.093	0.011
Sphaerobacter	0.03	0.015	0.102	0.155	0.039
Serratia	0.003	0.002	0.073	0.084	0.001
Flavitalea	0.136	0.047	0.202	0.117	0.036
Bacillariophyta	0.082	0.048	0.141	0.066	0.019
Parasegetibacter	0.071	0.046	0.13	0.089	0.028
Methyloceanibacter	0.096	0.039	0.152	0.06	0.01
Hyphomicrobium	0.062	0.029	0.116	0.06	0.004
Umezawaea	0.025	0.015	0.074	0.051	0
Pseudonocardia	0.053	0.019	0.1	0.048	0.001
Longilinea	0.04	0.022	0.086	0.051	0.002
Rhodococcus	0.027	0.012	0.073	0.038	0
Stenotrophomonas	0.011	0.009	0.056	0.043	0
Opitutus	0.065	0.041	0.107	0.047	0.038
Ochrobactrum	0.006	0.004	0.044	0.046	0.001
Armatimonadetes_gp4	0.095	0.026	0.133	0.062	0.026
Legionella	0.021	0.011	0.059	0.036	0
Aquicella	0.053	0.033	0.089	0.052	0.043
Proteus	0	0	0.034	0.046	0.002
Delftia	0.001	0.001	0.034	0.034	0
Chloroflexus	0.033	0.02	0.066	0.023	0.003
Fusobacterium	0.006	0.004	0.038	0.064	0.026
Chlorophyta	0.015	0.007	0.043	0.023	0
Cellulosimicrobium	0.01	0.007	0.039	0.062	0.039
Corynebacterium	0.003	0.003	0.032	0.035	0.001
Acinetobacter	0.021	0.01	0.049	0.029	0
Enterobacter	0.012	0.005	0.039	0.044	0.007
Paenibacillus	0.008	0.003	0.032	0.019	0
Actinomadura	0.016	0.006	0.039	0.032	0.003

Thermomarinilinea	0.014	0.011	0.036	0.028	0.004
Dongia	0.038	0.015	0.061	0.027	0.011
Achromobacter	0.006	0.005	0.028	0.014	0
Actinocorallia	0.016	0.01	0.038	0.031	0.006
Dactylosporangium	0.033	0.01	0.052	0.037	0.033
Oscillochloris	0.007	0.005	0.025	0.013	0
Gardnerella	0	0	0.017	0.02	0
Enhydrobacter	0.003	0.003	0.019	0.02	0.001
Holdemania	0	0	0.016	0.019	0
Herbidospora	0.009	0.005	0.023	0.029	0.035
Thermosporothrix	0.006	0.005	0.02	0.028	0.035
Staphylococcus	0.001	0.001	0.014	0.013	0
Georgfuchsia	0.006	0.006	0.019	0.017	0.003
Haemophilus	0.001	0.002	0.014	0.027	0.031
Granulicatella	0	0	0.012	0.02	0.008
Phyllobacterium	0.004	0.002	0.016	0.014	0.001
Methylobacterium	0.004	0.003	0.016	0.012	0
Kibdelosporangium	0.009	0.007	0.02	0.018	0.026
Candidatus Pelagibacter	0	0	0.011	0.014	0.001
Dyadobacter	0.002	0.003	0.012	0.012	0.001
Arcobacter	0.001	0.001	0.01	0.013	0.004
Asinibacterium	0.006	0.004	0.014	0.009	0.002
Peptostreptococcus	0	0	0.008	0.012	0.005
Cnuella	0.001	0.002	0.009	0.016	0.031
Kaistia	0.004	0.003	0.012	0.016	0.046
Psychrobacter	0	0	0.007	0.008	0
Bryobacter	0.006	0.003	0.013	0.015	0.046
Vampirovibrio	0.007	0.006	0.013	0.007	0.029
Campylobacter	0.002	0.002	0.008	0.013	0.029
Veillonella	0.002	0.002	0.008	0.013	0.046
Croceicoccus	0	0	0.006	0.009	0.005
Gracilibacillus	0	0	0.006	0.01	0.011
Telmatospirillum	0	0	0.006	0.008	0.002
Gp9	0	0	0.005	0.01	0.018
Deinococcus	0	0	0.005	0.004	0
Thiopseudomonas	0	0	0.005	0.011	0.037
Ferrovibrio	0.004	0.003	0.009	0.01	0.047
Coprobacter	0.001	0.001	0.006	0.007	0.003
Lentzea	0	0	0.005	0.006	0.001
Sneathia	0	0	0.005	0.009	0.019
Inhella	0	0	0.004	0.007	0.005
Nonomuraea	0.003	0.002	0.007	0.006	0.008
Pseudoalteromonas	0	0	0.004	0.005	0.001
Propionibacterium	0.003	0.003	0.007	0.007	0.036
Panacagrimonas	0.004	0.002	0.008	0.006	0.014
Actinobacillus	0	0	0.004	0.005	0.002
Amylibacter	0	0	0.004	0.006	0.002
Solimonas	0.006	0.003	0.01	0.007	0.034
Geothrix	0	0	0.004	0.008	0.023
Pediococcus	0.001	0.001	0.005	0.006	0.005
Atopobium	0	0	0.004	0.005	0.001
Anaerococcus	0	0	0.004	0.006	0.007
Vogesella	0	0	0.004	0.007	0.012
Acidobacterium	0	0	0.004	0.006	0.01

Sideroxydans	0.002	0.002	0.006	0.005	0.011
GpV	0.002	0.001	0.006	0.005	0.003
Methylobacillus	0.001	0.002	0.005	0.007	0.039
Turneriella	0.001	0.001	0.004	0.004	0.001
Verrucomicrobium	0.002	0.002	0.005	0.006	0.05
Alcaligenes	0	0	0.003	0.004	0.002
Gp13	0.001	0.001	0.004	0.004	0.002
Patulibacter	0	0.001	0.003	0.004	0.003
Solibacillus	0	0	0.003	0.003	0
Anaerobiospirillum	0	0	0.003	0.004	0.004
Ornatilinea	0.001	0.002	0.004	0.004	0.012
Insolitispirillum	0.002	0.001	0.004	0.004	0.019
Mucinivorans	0	0	0.003	0.003	0.001
Parvibaculum	0	0	0.002	0.004	0.007
Parvimonas	0	0	0.002	0.004	0.009
Morganella	0	0	0.002	0.003	0.004
Paraprevotella	0	0	0.002	0.004	0.014
Sinomonas	0.001	0.002	0.003	0.004	0.045
Colwellia	0	0	0.002	0.004	0.021
Solobacterium	0	0	0.002	0.004	0.014
Halopolyspora	0	0	0.002	0.003	0.005
Fictibacillus	0	0	0.002	0.004	0.027
Halanaerobium	0	0	0.002	0.004	0.036
Coxiella	0	0	0.002	0.003	0.002
Rheinheimera	0	0	0.002	0.003	0.023
Emticicia	0	0	0.002	0.004	0.049
Salicola	0	0	0.002	0.002	0.003
Succinivibrio	0	0	0.002	0.004	0.047
Hydrogenophaga	0	0	0.002	0.002	0.001
Brevinema	0	0	0.002	0.002	0.002
Cryptomonadaceae	0	0	0.002	0.003	0.023
GpVIII	0	0	0.002	0.002	0
Duganella	0	0	0.001	0.003	0.039
Phytomonospora	0	0	0.001	0.002	0.001
Catonella	0	0	0.001	0.003	0.01
Lewinella	0	0	0.001	0.002	0.006
Dehalogenimonas	0	0	0.001	0.003	0.013
Oceanobacillus	0	0	0.001	0.003	0.032
Cohnella	0	0	0.001	0.002	0.004
Dermabacter	0	0	0.001	0.003	0.032
Pelobacter	0	0	0.001	0.002	0.023
Cytophaga	0	0	0.001	0.002	0.025
Rhodoferax	0	0	0.001	0.002	0.038
Peptoniphilus	0	0	0.001	0.001	0.012
Mycoplasma	0	0	0.001	0.001	0.008
Saccharopolyspora	0	0	0.001	0.001	0.021
Thermoflavimicrobium	0	0	0.001	0.001	0.009
Pseudogulbenkiania	0	0	0.001	0.002	0.045
Azospira	0	0	0.001	0.001	0.009
Singulisphaera	0	0	0.001	0.001	0.016
Isosphaera	0	0	0.001	0.001	0.048
Aneurinibacillus	0	0	0.001	0.001	0.031
Denitratisoma	0.001	0.001	0	0	0.048
Albidovulum	0.002	0.001	0	0.001	0.047

Comamonas	0.004	0.004	0	0.001	0.048
Enterorhabdus	0.004	0.005	0	0	0.044
Vulcaniibacterium	0.005	0.003	0	0	0.003
Butyricicoccus	0.006	0.005	0	0	0.017
Mangrovibacterium	0.01	0.011	0	0	0.046
Intestinibacter	0.015	0.011	0.004	0.006	0.028
Acetatifactor	0.013	0.014	0	0.001	0.043
Hydrogenoanaerobacterium	0.014	0.014	0	0.001	0.036
Mizugakiibacter	0.033	0.019	0.015	0.014	0.042
Dorea	0.022	0.017	0.002	0.003	0.02
Marvinbryantia	0.02	0.018	0	0	0.023
Rhodoligotrophos	0.084	0.022	0.062	0.023	0.041
Allokutzneria	0.044	0.021	0.021	0.014	0.024
Ruminococcus	0.041	0.033	0.01	0.011	0.045
Intestinimonas	0.034	0.03	0	0	0.019
Eubacterium	0.035	0.032	0	0.002	0.026
Cupriavidus	0.051	0.04	0.012	0.009	0.036
Robinsoniella	0.042	0.038	0	0	0.024
Shewanella	0.044	0.043	0	0	0.028
Clostridium IV	0.062	0.051	0.01	0.023	0.031
Anaerorhabdus	0.057	0.046	0	0	0.014
Anaerotruncus	0.06	0.058	0	0.001	0.03
Methyloversatilis	0.182	0.038	0.116	0.046	0.002
Turicibacter	0.07	0.063	0.001	0.002	0.022
WPS-1_genera_incertae_sedis	0.115	0.039	0.043	0.029	0.001
Lactovum	0.082	0.066	0	0	0.013
Aeromonas	0.107	0.087	0.014	0.016	0.026
Dysgonomonas	0.104	0.09	0	0	0.018
Bifidobacterium	0.177	0.129	0.025	0.027	0.017
Blastococcus	0.411	0.16	0.22	0.112	0.016
Aquihabitans	0.723	0.15	0.516	0.155	0.008
Barnesiella	0.455	0.464	0.029	0.028	0.046
Bacteroides	0.631	0.476	0.126	0.339	0.028
Clostridium XlVb	0.608	0.579	0.001	0.003	0.028
Clostridium XlVa	0.723	0.677	0.055	0.089	0.035
Cetobacterium	1.604	1.339	0	0	0.016
