# Supplementary material for: Effects of Cultured Root and Soil Microbial Communities on the Disease of Nicotiana tabacum Caused by Phytophthora nicotianae
Source: Front Microbiol. 2020 May 15;11:929. doi: 10.3389/fmicb.2020.00929 (PMC7243367; doi:10.3389/fmicb.2020.00929)
Supplement: Supplementary file 8 [file Data_Sheet_8.pdf]

Table S4 Significantly (p-value < 0.05) different genera in S group and R group					
Genus	R: mean rel. freq. (%)	R: std. dev. (%)	S: mean rel. freq. (%)	S: std. dev. (%)	p-values
Acetanaerobacterium	0.007	0.006	0.001	0.002	0
Acetatifactor	0.1	0.081	0	0.001	0
Achromobacter	0.007	0.008	0.028	0.014	0
Aciditerrimonas	0.429	0.284	0.836	0.31	0
Actinobacillus	0	0	0.004	0.005	0.002
Actinocorallia	0.015	0.026	0.038	0.031	0.013
Actinomadura	0.01	0.015	0.039	0.032	0
Adhaeribacter	0.654	0.991	0.104	0.122	0.019
Aeromicrobium	0.112	0.087	0.167	0.076	0.031
Aeromonas	0.503	0.388	0.014	0.016	0
Aggregicoccus	0.015	0.015	0.045	0.022	0
Agromyces	0.158	0.16	0.435	0.277	0
Akkermansia	0.015	0.012	0.001	0.002	0
Alcaligenes	0	0	0.003	0.004	0.002
Algiphilus	0	0	0.003	0.004	0.001
Alistipes	0.09	0.054	0.02	0.022	0
Allobaculum	0.023	0.023	0.001	0.003	0
Allokutzneria	0.008	0.01	0.021	0.014	0.001
Alsobacter	0.012	0.012	0.041	0.026	0
Amycolatopsis	0.031	0.042	0.104	0.135	0.02
Amylibacter	0	0	0.004	0.006	0.002
Anaerobiospirillum	0	0	0.003	0.004	0.004
Anaerococcus	0	0	0.004	0.006	0.007
Anaeromyxobacter	0.06	0.072	0.145	0.096	0.002
Anaerorhabdus	0.33	0.316	0	0	0
Anaerostipes	0.079	0.059	0.01	0.01	0
Anaerotruncus	0.194	0.135	0	0.001	0
Anaerovorax	0.018	0.016	0	0	0
Ancylobacter	0.002	0.003	0.007	0.007	0.003
Aneurinibacillus	0	0	0.001	0.001	0.031
Anoxybacillus	0	0	0.004	0.007	0.025
Aquabacterium	0.012	0.014	0.043	0.025	0
Aquicella	0.022	0.021	0.089	0.052	0
Aquihabitans	0.362	0.253	0.516	0.155	0.022
Aquipuribacter	0.015	0.024	0	0	0.009
Aquisphaera	0.001	0.002	0.004	0.003	0
Arcobacter	0.001	0.003	0.01	0.013	0.004
Arenimonas	0.075	0.069	0.156	0.077	0.001
Aridibacter	0.157	0.122	0.276	0.124	0.002
Armatimonadetes_gp4	0.028	0.027	0.133	0.062	0
Armatimonadetes_gp5	0.024	0.025	0.082	0.057	0
Armatimonas/Armatimonadetes_gp1	0.039	0.057	0.01	0.007	0.033
Arthrobacter	0.569	0.396	1.215	0.712	0.001
Asaia	0.002	0.002	0	0	0.001
Asinibacterium	0.005	0.006	0.014	0.009	0.001
Atopobium	0.001	0.002	0.004	0.005	0.015
Bacillus	0.089	0.053	0.207	0.119	0
Bacteriovorax	0	0	0.001	0.001	0.007

Bacteroides	2.497	1.535	0.126	0.339	0
Barnesiella	1.934	1.347	0.029	0.028	0
Bauldia	0.019	0.024	0.038	0.021	0.005
Bdellovibrio	0.022	0.021	0.043	0.017	0.001
Bellilinea	0.002	0.005	0.011	0.012	0.003
Belnapia	0.047	0.074	0	0	0.008
Bifidobacterium	0.833	0.475	0.025	0.027	0
Bilophila	0	0	0.001	0.001	0.033
Blastocatella	0.081	0.056	0.197	0.084	0
Blastochloris	0.006	0.007	0.02	0.015	0.001
Blastomonas	0.001	0.002	0.005	0.005	0.003
Blautia	0.196	0.159	0.041	0.054	0
Bosea	0.029	0.022	0.074	0.041	0
Bradyrhizobium	0.261	0.233	0.595	0.264	0
BRC1_genera_incertae_sedis	0.002	0.003	0.012	0.01	0
Brevibacillus	0.002	0.003	0.011	0.012	0.001
Brevinema	0	0	0.002	0.002	0.002
Bryobacter	0.004	0.006	0.013	0.015	0.01
Buttiauxella	0.097	0.065	0.01	0.01	0
Butyricicoccus	0.034	0.024	0	0	0
Butyricimonas	0.002	0.003	0	0.001	0.032
Byssovorax	0.015	0.018	0.036	0.031	0.011
Caldilinea	0.031	0.027	0.129	0.059	0
Candidatus Koribacter	0.029	0.03	0.094	0.067	0
Candidatus Pelagibacter	0.002	0.003	0.011	0.014	0.007
Catellatospora	0.005	0.009	0.015	0.016	0.018
Catelliglobosispora	0.017	0.022	0.062	0.043	0
Catonella	0	0	0.001	0.003	0.01
Caulobacter	0.14	0.191	0.045	0.027	0.034
Cesiribacter	0.02	0.036	0	0	0.017
Cetobacterium	6.815	4.362	0	0	0
Chelativorans	0.142	0.236	0.008	0.016	0.016
Chitinibacter	0.003	0.005	0	0	0.012
Chitinophaga	0.174	0.173	0.704	0.343	0
Chloroflexus	0.013	0.014	0.066	0.023	0
Chlorophyta	0.015	0.017	0.043	0.023	0
Christensenella	0.008	0.007	0.001	0.003	0
Chryseobacterium	0.001	0.002	0.005	0.008	0.022
Chryseolinea	0.141	0.131	0.254	0.143	0.009
Chthonomonas/Armatimonadetes_gp3	0.047	0.047	0.24	0.138	0
Cloacibacillus	0.001	0.001	0	0	0.027
Clostridium III	0.002	0.003	0	0	0.003
Clostridium IV	0.157	0.092	0.01	0.023	0
Clostridium sensu stricto	0.564	0.32	0.088	0.062	0
Clostridium XlVa	2.831	1.863	0.055	0.089	0
Clostridium XlVb	5.213	3.199	0.001	0.003	0
Clostridium XVIII	0.102	0.079	0.006	0.012	0
Cnuella	0.002	0.003	0.009	0.016	0.044
Cohnella	0	0.001	0.001	0.002	0.021
Collinsella	0.004	0.007	0.001	0.002	0.031
Colwellia	0	0	0.002	0.004	0.021
Comamonas	0.013	0.015	0	0.001	0.001
Conexibacter	0.255	0.166	0.487	0.149	0
Constrictibacter	0.01	0.017	0	0	0.019

Coprobacillus	0.009	0.006	0	0	0
Coprococcus	0.049	0.038	0.002	0.003	0
Corallococcus	0.001	0.002	0.002	0.003	0.029
Corynebacterium	0.003	0.004	0.032	0.035	0.001
Coxiella	0	0.001	0.002	0.003	0.012
Craurococcus	0.005	0.007	0	0	0.005
Croceicoccus	0.001	0.004	0.006	0.009	0.039
Crossiella	0.002	0.004	0.006	0.007	0.031
Cryptosporangium	0.009	0.014	0	0	0.012
Dehalogenimonas	0	0	0.001	0.003	0.013
Delftia	0.003	0.004	0.034	0.034	0
Dermabacter	0	0	0.001	0.003	0.032
Desemzia	0.002	0.002	0	0	0
Desulfomonile	0	0.001	0	0	0.043
Devosia	0.151	0.129	0.285	0.267	0.039
Dialister	0.006	0.005	0.001	0.004	0.001
Dietzia	0.013	0.02	0	0	0.005
Diplorickettsia	0	0	0.003	0.005	0.006
Dongia	0.026	0.027	0.061	0.027	0
Dorea	0.071	0.05	0.002	0.003	0
Dyadobacter	0.005	0.007	0.012	0.012	0.02
Dysgonomonas	0.455	0.258	0	0	0
Elioraea	0.005	0.006	0.016	0.013	0.001
Elusimicrobium	0	0.001	0.002	0.002	0.021
Enhydrobacter	0.004	0.004	0.019	0.02	0.002
Enhygromyxa	0.002	0.003	0.008	0.006	0
Ensifer	0.043	0.047	0.157	0.093	0
Enterorhabdus	0.02	0.018	0	0	0
Erysipelotrichaceae_incertae_sedis	0.021	0.018	0	0.002	0
Eubacterium	0.114	0.073	0	0.002	0
Euzebya	0.1	0.148	0.005	0.004	0.008
Exiguobacterium	0.003	0.003	0.001	0.003	0.028
Faecalibacterium	0.539	0.371	0.076	0.138	0
Ferrovibrio	0.002	0.004	0.009	0.01	0.004
Fictibacillus	0	0	0.002	0.004	0.027
Filifactor	0.002	0.003	0	0.002	0.047
Filomicrobium	0.002	0.002	0	0	0.001
Fimbriimonas	0.001	0.002	0.006	0.006	0.001
Finegoldia	0.001	0.002	0	0	0.024
Flavitalea	0.091	0.087	0.202	0.117	0.001
Flavonifractor	0.161	0.104	0.042	0.119	0.001
Fluviicola	0.006	0.005	0.013	0.015	0.031
Fontimonas	0	0.001	0.003	0.004	0.016
Fretibacterium	0.001	0.001	0	0	0.017
Friedmanniella	0.104	0.173	0	0	0.011
Fulvivirga	0.002	0.004	0	0	0.045
Fusicatenibacter	0.054	0.056	0.012	0.024	0.004
Gaiella	2.447	2.032	5.397	1.542	0
Gardnerella	0	0	0.017	0.02	0
Gemmata	0.003	0.004	0.017	0.014	0
Gemmatimonas	2.223	1.664	5.195	1.059	0
Gemmiger	0.048	0.061	0.011	0.015	0.013
Geobacter	0.002	0.003	0.005	0.006	0.027
Geodermatophilus	0.082	0.127	0.017	0.017	0.029

Georgenia	0.006	0.011	0	0	0.014
Gp1	0.331	0.336	1.02	1.088	0.007
Gp10	0.119	0.134	0.564	0.238	0
Gp11	0.038	0.05	0.102	0.076	0.002
Gp12	0.002	0.005	0.006	0.007	0.028
Gp15	0.004	0.007	0.018	0.021	0.004
Gp16	0.929	0.735	1.909	0.688	0
Gp17	0.125	0.162	0.41	0.256	0
Gp18	0.02	0.028	0.059	0.048	0.002
Gp20	0.003	0.005	0.008	0.011	0.033
Gp25	0.017	0.023	0.048	0.025	0
Gp3	0.835	0.621	1.72	0.499	0
Gp4	0.954	1.027	3.161	1.682	0
Gp5	0.094	0.115	0.29	0.143	0
Gp6	1.454	1.202	4.222	1.335	0
Gp7	0.451	0.413	1.553	0.541	0
GpV	0.002	0.002	0.006	0.005	0.001
GpVIII	0	0	0.002	0.002	0
GpXIII	1.085	1.871	0.082	0.109	0.023
Gracilibacillus	0	0	0.006	0.01	0.011
Halanaerobium	0	0	0.002	0.004	0.036
Haliangium	0.039	0.044	0.081	0.065	0.014
Haliscomenobacter	0.001	0.002	0.004	0.005	0.006
Halopolyspora	0	0	0.002	0.003	0.005
Herbidospora	0.005	0.006	0.023	0.029	0.007
Herpetosiphon	0.016	0.016	0.048	0.031	0
Holdemanella	0.078	0.062	0.001	0.003	0
Holdemania	0	0	0.016	0.019	0
Hydrogenispora	0	0.001	0.002	0.002	0.006
Hydrogenoanaerobacterium	0.064	0.053	0	0.001	0
Hydrogenophaga	0	0	0.002	0.002	0.004
Hymenobacter	0.211	0.348	0.002	0.002	0.012
Hyphomicrobium	0.028	0.022	0.116	0.06	0
Ilumatobacter	0.388	0.259	0.975	0.338	0
Inhella	0	0.001	0.004	0.007	0.012
Intestinibacter	0.037	0.024	0.004	0.006	0
Intestinimonas	0.14	0.121	0	0	0
Intrasporangium	0.602	0.824	1.57	0.858	0
Isosphaera	0	0	0.001	0.001	0.048
Kaistia	0.002	0.003	0.012	0.016	0.009
Kibdelosporangium	0.002	0.006	0.02	0.018	0
Kitasatospora	0.022	0.016	0.083	0.049	0
Kofleria	0.452	0.391	1.029	0.379	0
Kribbella	0.084	0.072	0.206	0.136	0.001
Labilithrix	0.043	0.033	0.085	0.041	0
Labrys	0.012	0.013	0.041	0.019	0
Lachnospira	0.005	0.005	0.002	0.003	0.01
Lachnospiracea_incertae_sedis	0.441	0.323	0.009	0.015	0
Lacibacter	0.007	0.008	0.03	0.027	0
Lacibacterium	0.15	0.159	0.388	0.278	0.001
Lactobacillus	0.809	0.488	0.164	0.131	0
Lactonifactor	0.002	0.002	0	0	0.002
Lactovum	0.316	0.165	0	0	0
atescibacteria_genera_incertae_sedi	0.156	0.237	0.367	0.291	0.011

Lautropia	0.025	0.034	0.002	0.007	0.005
Lechevalieria	0.053	0.048	0.286	0.153	0
Legionella	0.009	0.008	0.059	0.036	0
Lentzea	0.001	0.001	0.005	0.006	0.004
Lewinella	0	0	0.001	0.002	0.006
Longilinea	0.015	0.015	0.086	0.051	0
Luteolibacter	0.007	0.01	0.019	0.017	0.01
Lysobacter	0.23	0.259	0.56	0.324	0.001
Mangrovibacterium	0.033	0.022	0	0	0
Marmoricola	0.323	0.219	0.596	0.319	0.002
Marvinbryantia	0.087	0.066	0	0	0
Megasphaera	0.003	0.003	0	0.001	0
Mesorhizobium	0.094	0.094	0.285	0.183	0
Methanomassiliicoccus	0.004	0.005	0.011	0.008	0.003
Methylobacillus	0.001	0.002	0.005	0.007	0.005
Methylobacterium	0.006	0.006	0.016	0.012	0.002
Methyloceanibacter	0.038	0.04	0.152	0.06	0
Methylocystis	0.008	0.008	0.003	0.005	0.025
Methyloparacoccus	0.001	0.003	0	0	0.035
Methylophilus	0.019	0.018	0.057	0.04	0
Methylopila	0.014	0.023	0	0	0.009
Methylotenera	0.005	0.008	0.021	0.024	0.006
Methyloversatilis	0.077	0.061	0.116	0.046	0.024
Microbacterium	0.062	0.108	0.22	0.267	0.013
Microlunatus	0.036	0.037	0.144	0.063	0
Microvirga	0.373	0.544	0.109	0.096	0.039
Minicystis	0.024	0.026	0.039	0.02	0.031
Mitsuaria	0.008	0.009	0.072	0.144	0.044
Mucinivorans	0	0	0.003	0.003	0.001
Mucispirillum	0.012	0.009	0	0.001	0
Mycobacterium	0.177	0.146	0.561	0.297	0
Neorhizobium	0.015	0.01	0.045	0.058	0.021
Niabella	0	0	0.001	0.001	0.011
Niastella	0.038	0.034	0.177	0.123	0
Nitriliruptor	0.003	0.006	0	0	0.039
Nitrososphaera	0.982	0.91	2.029	0.887	0
Nitrosospira	0.075	0.065	0.157	0.098	0.002
Nitrospira	0.359	0.311	0.95	0.216	0
Nitrospirillum	0.002	0.003	0.004	0.005	0.033
Nocardia	0.004	0.007	0.016	0.017	0.003
Nocardioides	0.564	0.4	1.398	0.857	0
Nonomuraea	0.001	0.002	0.007	0.006	0
Noviherbaspirillum	0.05	0.08	0.01	0.012	0.034
Novosphingobium	0.042	0.036	0.079	0.032	0.001
Oceanobacillus	0	0	0.001	0.003	0.032
Ochrobactrum	0.01	0.02	0.044	0.046	0.003
Ohtaekwangia	0.117	0.108	0.808	1.399	0.027
Opitutus	0.033	0.032	0.107	0.047	0
Ornatilinea	0	0.001	0.004	0.004	0
Ornithinimicrobium	0.009	0.013	0	0	0.007
Oryzihumus	0	0.001	0.004	0.006	0.011
Oscillibacter	0.719	0.523	0.008	0.009	0
Oscillochloris	0.009	0.01	0.025	0.013	0
Paenibacillus	0.008	0.007	0.032	0.019	0

Panacagrimonas	0.002	0.002	0.008	0.006	0
Parabacteroides	0.682	0.436	0.23	0.941	0.046
Paracoccus	0.02	0.019	0.008	0.006	0.01
Parafilimonas	0.01	0.01	0.03	0.017	0
Parasegetibacter	0.032	0.036	0.13	0.089	0
Parvibaculum	0	0.001	0.002	0.004	0.027
Parvimonas	0	0.001	0.002	0.004	0.019
Pedomicrobium	0.095	0.125	0.252	0.116	0
Pelobacter	0	0	0.001	0.002	0.023
Pelomonas	0.009	0.009	0.034	0.027	0
Peptococcus	0.682	0.449	0.066	0.314	0
Peptostreptococcus	0	0	0.008	0.012	0.005
Permianibacter	0.001	0.001	0	0	0.026
Phaeodactylibacter	0.023	0.027	0.124	0.106	0
Phascolarctobacterium	0.107	0.07	0.002	0.003	0
Phaselicystis	0.059	0.047	0.09	0.044	0.027
Phenylobacterium	0.144	0.114	0.273	0.088	0
Phycicoccus	0.114	0.103	0.338	0.27	0.001
Phycisphaera	0	0.001	0.001	0.002	0.025
Phytomonospora	0	0.001	0.001	0.002	0.027
Pilimelia	0.002	0.003	0.005	0.004	0.004
Pirellula	0	0.001	0.005	0.007	0.001
Piscinibacter	0.011	0.013	0.033	0.022	0
Planococcus	0.055	0.096	0	0	0.016
Planomicrobium	0.002	0.004	0	0	0.017
Plesiomonas	0.004	0.004	0	0.001	0
Polaromonas	0.01	0.013	0.035	0.032	0.002
Polyangium	0.009	0.008	0.018	0.012	0.006
Povalibacter	0.102	0.088	0.251	0.125	0
Prevotella	0.477	0.309	0.041	0.061	0
Prosthecomicrobium	0.001	0.002	0.003	0.003	0.02
Proteus	0.002	0.005	0.034	0.046	0.003
Pseudoalteromonas	0	0	0.004	0.005	0.001
Pseudoduganella	0.036	0.041	0.113	0.065	0
Pseudoflavonifractor	0.39	0.269	0.018	0.065	0
Pseudogulbenkiania	0	0	0.001	0.002	0.045
Pseudolabrys	0.127	0.162	0.269	0.158	0.005
Pseudonocardia	0.037	0.024	0.1	0.048	0
Pseudorhodoferax	0.028	0.013	0.002	0.005	0
Pseudoxanthomonas	0.069	0.091	0.019	0.023	0.023
Psychrilyobacter	0.003	0.005	0.001	0.002	0.041
Psychrobacter	0.001	0.002	0.007	0.008	0.003
Ramlibacter	0.137	0.146	0.253	0.081	0.003
Reyranella	0.16	0.17	0.318	0.123	0.001
Rheinheimera	0	0	0.002	0.003	0.023
Rhizobacter	0.01	0.009	0.02	0.012	0.003
Rhizobium	0.044	0.029	0.133	0.143	0.007
Rhizocola	0.012	0.014	0.058	0.056	0.001
Rhizorhapis	0.01	0.018	0.043	0.037	0.001
Rhodococcus	0.04	0.026	0.073	0.038	0.002
Rhodocytophaga	0.327	0.542	0.006	0.009	0.013
Rhodoligotrophos	0.04	0.033	0.062	0.023	0.016
Rhodomicrobium	0.015	0.022	0.003	0.005	0.019
Rhodoplanes	0.195	0.189	0.731	0.277	0

Robinsoniella	0.183	0.141	0	0	0
Romboutsia	0.087	0.057	0.032	0.057	0.003
Roseburia	0.7	0.408	0.029	0.019	0
Roseimicrobium	0.003	0.005	0.008	0.006	0.007
Roseomonas	0.141	0.205	0.009	0.007	0.008
Rubellimicrobium	0.475	0.767	0.008	0.007	0.011
Rubricoccus	0.004	0.006	0	0	0.002
Rubritepida	0.02	0.033	0	0	0.013
Rubrobacter	0.314	0.496	0.029	0.019	0.016
Rufibacter	0.059	0.097	0	0	0.011
Rugosimonospora	0.031	0.043	0.077	0.084	0.028
Ruminococcus	0.191	0.116	0.01	0.011	0
Ruminococcus2	0.042	0.048	0.006	0.011	0.003
ccharibacteria_genera_incertae_sed	0.197	0.099	0.41	0.196	0
Saccharopolyspora	0	0	0.001	0.001	0.021
Salicola	0	0.001	0.002	0.002	0.028
Sediminibacterium	0.009	0.013	0.022	0.018	0.007
Segetibacter	0.117	0.192	0.022	0.041	0.036
Selenomonas	0.001	0.002	0	0	0.025
Serratia	0.003	0.004	0.073	0.084	0.001
Shewanella	0.11	0.068	0	0	0
Shimazuella	0	0	0.003	0.007	0.026
Sideroxydans	0.001	0.002	0.006	0.005	0
Singulisphaera	0	0	0.001	0.001	0.016
Smaragdicoccus	0.002	0.003	0.009	0.007	0.001
Sneathia	0	0	0.005	0.009	0.019
Solibacillus	0.001	0.001	0.003	0.003	0.003
Solimonas	0.002	0.004	0.01	0.007	0
Solirubrobacter	0.412	0.288	2.209	0.821	0
Solobacterium	0	0	0.002	0.004	0.014
partobacteria_genera_incertae_sedi	0.331	0.327	0.76	0.487	0.001
Sphaerobacter	0.029	0.023	0.102	0.155	0.035
Sphingobium	0.078	0.099	0.225	0.278	0.025
Sphingomonas	2.352	1.79	5.229	1.846	0
Sphingopyxis	0.007	0.01	0.04	0.042	0.001
Spirosoma	0.003	0.005	0	0	0.024
Sporichthya	0.018	0.015	0.036	0.026	0.005
Sporocytophaga	0.407	0.666	0.001	0.002	0.011
Sporosarcina	0.009	0.009	0.018	0.012	0.005
Stenotrophomonas	0.008	0.009	0.056	0.043	0
Stomatobaculum	0.213	0.134	0.001	0.002	0
Streptobacillus	0.003	0.004	0	0.002	0.014
Streptomyces	0.164	0.129	0.445	0.198	0
Streptophyta	0.026	0.013	0.115	0.061	0
Subdivision3_genera_incertae_sedis	0.214	0.198	0.764	0.345	0
Succinivibrio	0	0	0.002	0.004	0.047
Sutterella	0.16	0.098	0.001	0.003	0
Tahibacter	0.003	0.004	0.019	0.018	0
Telmatospirillum	0	0	0.006	0.008	0.002
Terrabacter	0.23	0.271	0.68	0.391	0
Terrimicrobium	0.002	0.003	0.006	0.006	0.016
Terrimonas	0.133	0.133	0.516	0.203	0
Thauera	0.002	0.003	0	0	0.012
Thermoflavimicrobium	0	0	0.001	0.001	0.009

Thermogutta	0	0	0	0.001	0.011
Thermoleophilum	0.306	0.247	0.923	0.31	0
Thermomarinilinea	0.013	0.014	0.036	0.028	0.001
Thermosporothrix	0.006	0.008	0.02	0.028	0.038
Truepera	0.07	0.109	0.006	0.007	0.014
Turicibacter	0.456	0.304	0.001	0.002	0
Turneriella	0.001	0.002	0.004	0.004	0.006
Umezawaea	0.011	0.012	0.074	0.051	0
Unclassified	32.144	4.915	26.359	3.008	0
Vampirovibrio	0.002	0.004	0.013	0.007	0
Variovorax	0.004	0.004	0.012	0.011	0.003
Verrucomicrobium	0.001	0.002	0.005	0.006	0.012
Vibrio	0.007	0.005	0	0.001	0
Virgisporangium	0.019	0.017	0.031	0.021	0.044
Vogesella	0	0.001	0.004	0.007	0.023
Zavarzinella	0.003	0.004	0.016	0.017	0.001
