# Supplementary material for: Effects of Cultured Root and Soil Microbial Communities on the Disease of Nicotiana tabacum Caused by Phytophthora nicotianae
Source: Front Microbiol. 2020 May 15;11:929. doi: 10.3389/fmicb.2020.00929 (PMC7243367; doi:10.3389/fmicb.2020.00929)
Supplement: Supplementary file 9 [file Data_Sheet_9.pdf]

Table S5 Topological properties of the empirical pMENs of soil bacterial communities in nine groups.

Network Indexes	CK_30	CK_60	CK_90	R1_30	R1_60	R1_90	S1_30	S1_60	S1_90
Total OTU	4465	4723	5244	4843	4671	3699	4173	5825	5140
Number of OTU with 8 in all 8 samples	1072	1111	1064	1135	1137	288	1021	1775	1176
cutoff	0.96	0.96	0.97	0.96	0.96	0.98	0.96	0.99	0.95
Total nodes	485	443	554	525	497	220	442	357	572
Total links	1560	1125	1392	1682	1280	668	782	1720	1371
Positive links	575	464	771	802	551	644	394	423	546
R square of power-law	0.912	0.824	0.889	0.873	0.899	0.783	0.914	0.618	0.837
module	47	61	49	67	70	18	52	22	63
modularity	0.502	0.525	0.688	0.538	0.609	0.746	0.686	0.404	0.589
Average degree (avgK)	6.433	5.079	5.025	6.408	5.151	6.073	3.538	9.636	4.794
Average clustering coefficient (avgCC)	0.151	0.129	0.204	0.153	0.149	0.402	0.124	0.121	0.177
Average path distance (GD)	4.438	5.152	6.277	4.357	4.327	5.809	4.968	4.149	5.689
