# Supplementary material for: Effects of Cultured Root and Soil Microbial Communities on the Disease of Nicotiana tabacum Caused by Phytophthora nicotianae
Source: Front Microbiol. 2020 May 15;11:929. doi: 10.3389/fmicb.2020.00929 (PMC7243367; doi:10.3389/fmicb.2020.00929)
Supplement: Supplementary file 10 [file Data_Sheet_10.pdf]

Table S6 Inhibition experiments and identifications of isolated functional strains

Strain	Isolated sample	Inhibition zone (cm)		Identification	Similarity
		1st experiment	2nd experiment		
Strain 1	Root	1.3	0.8	*Acinetobacter calcoaceticus*	99%
Strain 2	Root	1.1	1	*Acinetobacter calcoaceticus*	99%
Strain 3	Root	1.6	1.4	*Acinetobacter calcoaceticus*	99%
Strain 4	Root	1.5	1.1	*Acinetobacter calcoaceticus*	99%
Strain 5	Root	1.4	1.1	*Acinetobacter calcoaceticus*	99%
Strain 6	Root	1.2	0.8	*Acinetobacter calcoaceticus*	99%
Strain 7	Root	1	1	*Acinetobacter calcoaceticus*	99%
Strain 8	Root	1.5	1.2	*Acinetobacter calcoaceticus*	99%
Strain 9	Soil	0.8	0.5	*Acinetobacter calcoaceticus*	99%
Strain 10	Soil	0.9	0.8	*Acinetobacter calcoaceticus*	99%
Strain 11	Soil	1.1	0.7	*Acinetobacter calcoaceticus*	99%
Strain 12	Soil	1.1	0.7	*Acinetobacter calcoaceticus*	99%
Strain 13	Root	0.9	0.6	*Citrobacter amalonaticus*	99%
Strain 14	Root	1.1	0.6	*Citrobacter amalonaticus*	99%
Strain 15	Root	0.9	0.9	*Enterobacter cloacae*	99%
Strain 16	Soil	1.1	0.6	*Bacillus sp.*	99%
Strain 17	Root	0.9	0.6	*Stenotrophomonas maltophilia*	99%
Strain 18	Soil	0.4	0.3	*Stenotrophomonas maltophilia*	99%
