# Supplementary figures and images for: Effects of Cultured Root and Soil Microbial Communities on the Disease of Nicotiana tabacum Caused by Phytophthora nicotianae
Source: Front Microbiol. 2020 May 15;11:929. doi: 10.3389/fmicb.2020.00929 (PMC7243367; doi:10.3389/fmicb.2020.00929)

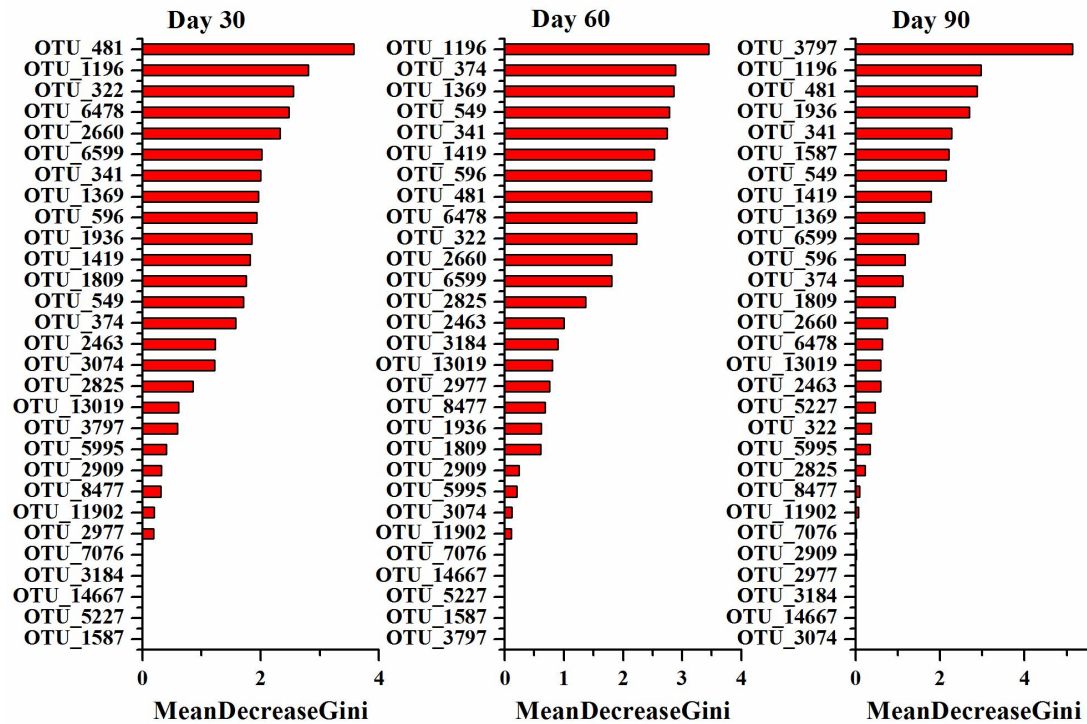

Fig. S5 The importance of 29 functional OTUs base on random forest analysis on day 30, 60, and 90, respectively

Supplement: Supplementary file 11 [file Data_Sheet_11.PDF]
