# Supplementary material for: Effects of Cultured Root and Soil Microbial Communities on the Disease of Nicotiana tabacum Caused by Phytophthora nicotianae
Source: Front Microbiol. 2020 May 15;11:929. doi: 10.3389/fmicb.2020.00929 (PMC7243367; doi:10.3389/fmicb.2020.00929)
Supplement: Supplementary file 12 [file Data_Sheet_12.PDF]

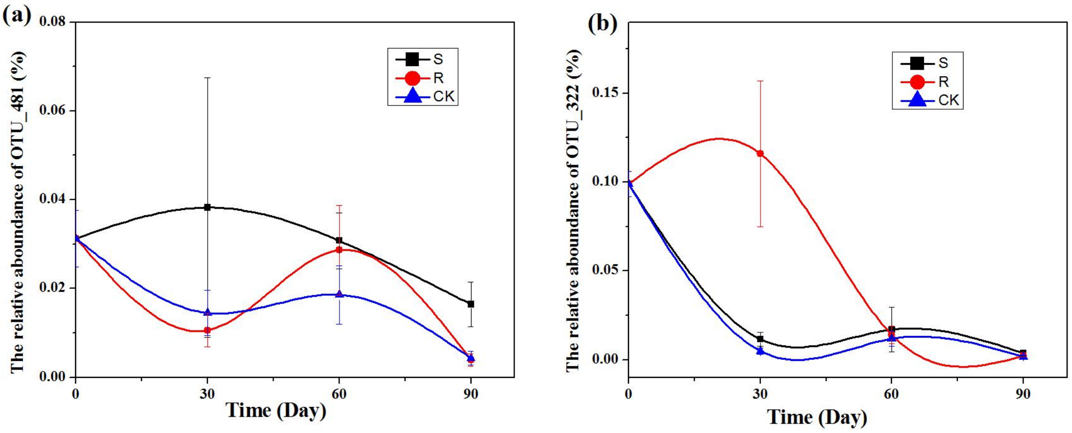

Fig. S6 Relative abundance of Two OTUs over time: (a) OTU_481, (b) OTU_322. CK: control group; R: treatment with root functional microflora; S: treatment with soil functional microflora.
